# Modification of the dermal matrix by senescence associated lipids and its functional consequence

**DOI:** 10.1016/j.redox.2026.104069

**Published:** 2026-02-10

**Authors:** Sarah Jelleschitz, Christopher Kremslehner, Ionela-Mariana Nagelreiter, Michael Mildner, Melanie Salek, Christina Bauer, Alexandra Stiegler, Adrian Sandgren Fors, Michaela Schirato, Christian Freystätter, Agnès Tessier, Francesca Marcato, Gaëlle Gendronneau, Nada André, Youcef Ben Khalifa, Zhixu Ni, Maria Fedorova, Olga Oskolkova, Marie-Sophie Narzt, Florian Gruber

**Affiliations:** a Department of Dermatology, Medical University of Vienna, Austria; b CDL SKINMAGINE, Vienna, Austria; c Center for Brain Research, Medical University of Vienna, Austria; d Department of Plastic, Reconstructive and Aesthetic Surgery, Medical University of Vienna, Austria; e Chanel Parfums Beauté, Pantin, France; f Center of Membrane Biochemistry and Lipid Research, University Hospital and Faculty of Medicine Carl Gustav Carus of TU Dresden, Germany; g Institute of Pharmaceutical Sciences, University of Graz, Austria; h Ludwig Boltzmann Institute for Traumatology, AUVA Research Center, Linz/Vienna, Austria

## Abstract

Senescent dermal fibroblasts accumulate and secrete chemically reactive lipids that are components of the senescence-associated secretory phenotype (SASP). These lipids, including 4-hydroxynonenal (HNE) and reactive oxidized phospholipids (OxPL), covalently bind to and modify proteins via Schiff base formation or Michael adduction. Our study examined lipid-induced collagen modifications and their impact on skin cells to evaluate the long-term consequences of senescent cells on the tissue microenvironment. Using mass spectrometry and biochemical analyses, we identified both high and low molecular-weight modifications to collagen types I, II and IV. Collagen modified by HNE reduced fibroblast proliferation and induced stress responses. In contrast, collagen modified by OxPL provoked inflammatory signaling. Both types of modifications influenced matrix remodeling by increasing proteinase expression while reducing collagen expression. Modified collagen also elevated levels of intracellular reactive oxygen species and lipid peroxidation. Macrophages cultured on modified collagen displayed altered cytokine profiles and Toll-like receptor signaling impairment, that depended on the specific type of lipid modification. Similarly, keratinocytes exposed to modified basal lamina collagen IV showed transient stress responses, increased cytokine expression, and reduced matrix metalloproteinase expression. Furthermore, lipid-modified collagen incorporated into organotypic skin equivalents disturbed keratinocyte differentiation and elevated markers of cellular senescence. These skin models also showed reduced epidermal thickness with HNE-modified collagen and parakeratosis on OxPL-modified matrices. In conclusion, the findings suggest that SASP lipids secreted by senescent fibroblasts alter collagen structure and the fate of residing cells. The responses are likely caused by cell-associated oxidation events upon interaction of cells with a lipid-modified matrix and can be inhibited by antioxidants in macrophages. Given collagen's long half-life in tissues, these modifications may represent a persistent mechanism by which senescent cells affect the tissue microenvironment beyond the lifespan of soluble SASP factors ― thereby sustaining an aged phenotype over extended periods.

## Highlights

• Reactive lipid aldehydes accumulating in and secreted by senescent cells, covalently adduct to the extracellular matrix proteins collagen types I, II, and IV.
• Fibroblasts cultured on lipid modified collagen display elevated levels of cytokines, altered matrix turnover and an early senescence phenotype.
• Monocyte derived cells grown on modified matrix show altered expression of cyto-/chemokines, elevated senescence markers and impaired pattern recognition receptor signaling.
• Organotypic skin equivalents with modified collagen display disturbed epidermal differentiation and elevated expression of senescence markers.

## Graphical abstract

Graphic created with Procreate (Apple). Illustrative elements adapted from Servier Medical Art (https://smart.servier.com/), licensed under CC BY 4.0.

**Figure:**
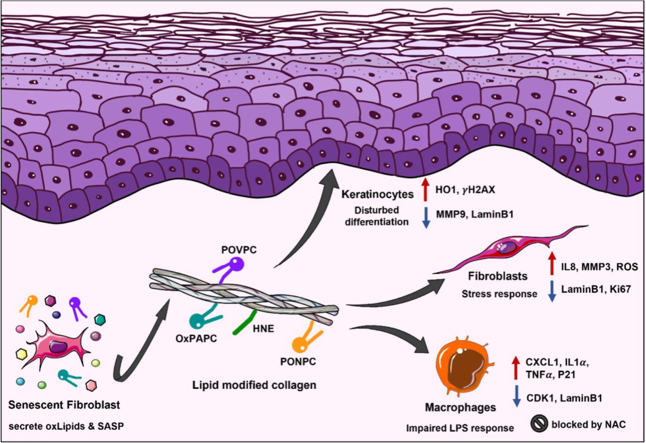

## 1 Introduction

The human skin serves as a critical barrier that protects against infections, dehydration, and withstands mechanical stress. The complex organ is composed of multiple layers, with the epidermis - primarily made up of keratinocytes (KC) - forming the outermost layer. As keratinocytes migrate from the proliferative stratum basale, they undergo a terminal differentiation program upon detachment from the basal membrane. Through this process, keratinocytes cease proliferation, lose their nuclei, and are eventually shed from the skin, thereby maintaining a continuously renewing and dynamic barrier [1].

The dermis lies between the epidermis and the subcutaneous tissue and contains a fibrous structure composed of collagens, elastic fibers, and other macromolecules collectively known as the extracellular matrix (ECM). Fibroblasts (FB), the predominant cell type of the dermis, can be classified into papillary and reticular phenotypes and are responsible for synthesizing ECM components. Collagen types I and III are primarily found in the dermis, while collagen type IV is localized in the basement membrane of the epidermis. With aging, degradation or modification of collagen contributes to visible aging signs such as wrinkles and skin sagging, while age spots, are associated with lipofuscin accumulation [[2], [3], [4]]. Skin aging results from intrinsic factors - such as cumulative damage and replicative exhaustion in cells - as well as extrinsic factors, with environmental influences like UV irradiation, air pollution, and oxidative stress playing a particularly significant role. Both, intrinsic and extrinsic factors contribute to the accumulation of senescent cells in aged skin [5,6]. Cellular senescence is a state characterized by a prolonged and generally irreversible cell cycle arrest, macromolecular damages, altered metabolism and a high secretion of a broad array of inflammatory and proteolytic factors collectively referred as the senescence-associated secretory phenotype (SASP). Senescent cells can be detected in situ through approaches that integrate multiple diagnostic markers [7]. The SASP includes expression and secretion of various proteins such as cytokines, chemokines, and proteases [8]. As previously reported, oxidized lipids also contribute to this phenotype [9].

Senescent dermal fibroblasts accumulate oxidized phospholipids in a cell-associated manner enabling hydrophilic moieties to either face outward [10] or be secreted, for instance, as membrane components of extracellular vesicles [11]. In addition to lysophospholipids, several lipid species containing reactive aldehyde groups - classified as epilipids - have been found elevated in senescent fibroblasts [12]. Furthermore, senescent cells show increased lipid droplet formation and accumulation of lipid aldehydes [13]. Reactive lipid aldehydes can covalently modify biomolecules [14,15], primarily through Schiff base formation and Michael addition. Schiff base formation results from reactions between amino groups, such as ε-amino groups of lysine residues and aldehyde or ketone groups from oxidized lipids [16]. In contrast, Michael additions involve α,β-unsaturated carbonyls acting as electrophilic Michael acceptors, forming new carbon-carbon bonds with Michael donors like nucleophilic cysteine thiols and secondary amines of lysine, histidine, and arginine residues [17].

Notably, the oxidized phospholipids palmitoyl-oxononanoyl phosphatidylcholine (PONPC) and palmitoyl-oxovaleroyl phosphatidylcholine (POVPC), which we observed to accumulate and be secreted by senescent cells [12], can form Schiff bases with lysines. POVPC additionally forms Michael adducts with lysines, histidines, and cysteines [18]. Among lipid peroxidation products, 4-hydroxynonenal (HNE) stands out as highly reactive; besides generating Schiff bases and Michael adducts, it also induces protein crosslinking [19,20]. In our study, we incorporated a more complex model system: autoxidized 1-palmitoyl-2-arachidonoyl-*sn*-glycero-3-phosphocholine (OxPAPC), yielding a well-studied array of oxidation products. These include lipid aldehydes derived from the oxidation of its arachidonic acid moiety [21,22].

In this study, we investigated whether reactive oxidized lipids identified in senescent cells can covalently modify collagen - the most abundant fibrous structural protein in the ECM. Furthermore, we examined the biological responses regarding inflammation, stress and propagation of senescence of resident skin cells (FB, KC, macrophages) to aldehyde-modified collagen, in comparison to their responses to unmodified collagen. Our findings suggest that, through this mechanism, the SASP could elicit long-term negative effects on the ECM that are compatible with models of how senescence-associated phenotypes are locally propagated in tissues, including the skin [23,24].

## 2 Materials and methods

### 2.1 Primary cells and cell culture conditions

All skin samples for biopsies and cell isolation used in this study were acquired under approval by the Ethics Committee of the Medical University of Vienna [ECS 1969/2021]. Written informed consent was obtained from all subjects. All primary cells used in this study were isolated from abdominoplasty tissue obtained from individuals diagnosed with cutis laxa. Fibroblasts were obtained from four donors (27-year-old male, 34-year-old male, 32-year-old female, and 41-year-old female). Keratinocytes were isolated from two donors (38-year-old female and 27-year-old male). Skin equivalents were generated using fibroblasts from 34-year-old female, 42-year-old female, and 25-year-old male donors, combined with keratinocytes from 49-year-old female, 35-year-old female, and 30-year-old female donors, respectively.

Human dermal fibroblasts were isolated and cultured in Dulbecco's Modified Eagle Medium (DMEM/F-12 + GlutaMAX Gibco, USA) supplemented with 10% of heat-inactivated fetal bovine serum, (FBS, Sigma-Aldrich, USA) and 1% Penicillin Streptomycin (Gibco, Thermo Fisher, USA). Primary keratinocytes were obtained from patient epidermis and cultured in DermaLife growth medium (Lifeline Cell Technology, USA) supplemented with LifeFactors. Since it is known that monocyte derived macrophages migrate into aged skin [23] we further selected THP-1 cells for our experiments. The widely studied human monocytic cell line isolated from the blood of a leukemia patient was grown in RPMI Medium 1640 + GlutaMAX (Gibco, USA) supplemented with 10% of heat-inactivated FBS and antibiotic-antimycotic solution (Gibco, Thermo Fisher, USA). The THP-1 cells were grown at a density of 0.2 – 1 × 10^6^ cells/ml. All cells were cultured at 37 °C in a humidified atmosphere containing 5% CO_2_.

### 2.2 Thin layer chromatography

OxPAPC was generated from 1-palmitoyl-2-arachidonyl-*sn*-glycero-3-phosphorylcholine (PAPC, Avanti Polar Lipids, USA) by autoxidation [21]. Aliquots of 600 μg PAPC dried as a thin film in 4 ml glass vials were oxidized under compressed ambient air for five days in darkness and thereafter stored at −20 °C under argon. 1-Palmitoyl-2-(5′-oxo-valeroyl)-*sn*-glycero-3-phosphocholine (POVPC) and 1-palmitoyl-2-(9′-oxo-nonanoyl)-*sn*-glycero-3-phosphocholine (PONPC, both from Avanti Polar Lipids, USA) were stored as chloroform solutions at −20 °C. Aliquots of these solutions were dried in 4 ml glass vials under the stream of argon. Ethanolic solution of HNE (Merck Millipore Corp., USA) was evaporated in a glass vial. The dried lipids (100 μg each) were emulgated by vigorous vortexing in 100 μl Dulbecco's Phosphate Buffered Saline (DPBS, Gibco, USA), and 1 mg sodium borohydride (NaBH_4_, Merck KGaA, Germany) was added to reduce aldehydes and ketones to respective alcohol groups. After 30 min incubation on ice, 0.7 M aqueous formic acid (Honeywell, Fluka, Germany) was added to the lipid mix to obtain pH 3.0. Subsequently, 1 ml chloroform was added, and after phase separation the lower phase was transferred into a new glass vial. Samples were evaporated under the stream of argon and then re-dissolved in chloroform (or ethanol for HNE) to obtain a 1 μg/μl solutions. DPPC (Avanti Polar Lipids, USA) and PAPC were used as controls. Oxidized or reduced lipids (10 μg each) were applied onto a 20 × 20 cm TLC silica gel 60W plate on aluminum foil (Merck KGaA, Germany) using a Hamilton syringe (Sigma-Aldrich, USA). After drying on air, the TLC plate was developed in vertical TLC chambers with a first liquid phase of chloroform/methanol/water 100:50:10 (by vol.) up to 10 cm and then with methanol/chloroform/water 65:25:4 (by vol.) as a second liquid phase. After drying on air, aldehydes were visualized by dipping the plate into Schiff's reagent (Sigma-Aldrich, USA) yielding magenta-colored spots [25].

### 2.3 Collagen modification with lipids

The required amount of lipid was dried in a glass vial under the stream of argon to obtain final concentrations of 100 μg/ml HNE, 600 μg/ml OxPAPC, 180 μg/ml PONPC and 180 μg/ml POVPC in aqueous buffer and collagen solution. In detail, for the modification reaction, the dried lipids were redissolved in three parts of K_2_HPO_4_ buffer, consisting of 50 mM K_2_HPO_4_ · 3H_2_O and 200 mM KCl and adjusted with 1 M HCl to a final pH of 7.4 as described previously [26]. One part of PureCol Type I bovine collagen solution (3.1 mg/ml, Advanced BioMatrix, Sweden) was added to the buffered lipid suspensions to obtain a final collagen concentration of 0.75 mg/ml. For the modification reaction, the buffered lipid-collagen mixtures were incubated for 4h at 37 °C based on an adapted POVPC-BSA modification protocol [27,28]. When using collagen from human placenta (Bornstein and Traub Type IV, Sigma-Aldrich, USA), dissolved in 3.33 ml of 0.5 M acetic acid to a concentration of 1.5 mg/ml, two parts of buffer and two parts of collagen were used to obtain the same collagen end concentration of 0.75 mg/ml.

### 2.4 Detection of carbonylated proteins by dot-blot

Collagen was modified using different concentrations of lipids with the method described above. Thereafter the collagen samples (1 μl each) were applied as quadruplicate dots onto a nitrocellulose membrane (Bio-Rad, USA) using a micro syringe. After drying the samples, the membrane was washed with water and PBS/20% methanol by vol., and then the membrane was incubated for 5 min in 2 M HCl. Subsequently, the membranes were incubated for 10 min in a 10 mM 2,4-dinitrophenylhydrazine phosphoric acid solution (DNPH, Supelco, India) diluted in 2 M HCl [29]. DNPH reacts with the protein carbonyls under acidic conditions and forms stable 2,4-dinitrophenylhydrazones (DNP), detectable with *anti*-DNP anti-rabbit antibody (D9656, Sigma-Aldrich, USA) used in a dilution of 1:10 000 in 2% BSA (w/v) in PBS followed by a secondary anti-rabbit IgG-HRP (Bio-Rad 1706515, 1:10 000).

### 2.5 LC-MS/MS analysis of lipid modified collagen

Lipid modified collagen was mixed with Laemmli sample buffer, separated by sodium dodecyl sulfate polyacrylamide gel electrophoresis (SDS-PAGE, 12%T, 1 mm, 200 V; Bio-Rad mini protean III cell; Bio-Rad Laboratories GmbH, Germany), and stained with Coomassie Brilliant Blue G-250. Protein bands were excised, destained with 50% (v/v) acetonitrile in 50 mmol/L NH_4_HCO_3_ (1 h, 37 °C, 750 rpm), dehydrated with 100% acetonitrile and dried under vacuum. Proteins were digested with trypsin in 3 mM NH_4_HCO_3_ (250 ng, 4 h, 37 °C, 550 rpm), and peptides obtained were extracted using consecutive incubations with 100%, 50% (v/v; aqueous solution) and 100% acetonitrile with 15 min sonication for each step. Combined extracts were concentrated under vacuum. Peptides were dissolved in 0.5% formic acid in aq. 60% acetonitrile and further diluted 1:10 (v/v) with 0.1% formic acid in 3% aqueous acetonitrile. Samples were analyzed by UPLC-MS [30]. A nanoAcquity UPLC (Waters GmbH, Germany) was coupled online to an LTQ-Orbitrap XL ETD mass-spectrometer equipped with a nano-ESI source (Thermo Fischer Scientific, Germany). Eluent A was aqueous formic acid (0.1% v/v), and eluent B was formic acid (0.1% v/v) in acetonitrile. Samples (10 μl) were loaded onto the trap column (nanoAcquity symmetry C18, internal diameter 180 μm, length 20 mm, particle diameter 5 μm) at a flow rate of 10 μl/min. Peptides were then separated on a BEH 130 column (C18-phase, internal diameter 75 μm, length 100 mm, particle diameter 1.7 μm) with a flow rate of 0.4 μl/min. Several linear gradients were used consequently: from 3% to 9% (2.1 min), 9.9% (1.9 min), 17.1% (10 min), 18% (0.5 min); 20.7% (0.2 min), 22.5% (3.1 min), 25.6% (3 min), 30.6% (5 min), 37.8% (2.8 min) and finally to 81% eluent B (2 min). Together with an equilibration time of 12 min the samples were injected every 46 min. The transfer capillary temperature was set to 200 °C and the tube lens voltage to 120 V. An ion spray voltage of 1.5 kV was applied to a PicoTip online nano-ESI emitter (New Objective, Germany). The precursor ion survey scans were acquired at an orbitrap (resolution of 60 000 at m/z 400) for a *m*/*z* range from 400 to 2000. CID-tandem mass spectra (isolation width 2, activation Q 0.25, normalized collision energy 35%, activation time 30 ms) were recorded in the linear ion trap by data-dependent acquisition (DDA) for the top six most abundant ions in each survey scan with dynamic exclusion for 60 s using Xcalibur software (version 2.0.7). The acquired tandem mass-spectra were searched against the UniProt database using Sequest search engine (Proteome Discoverer 1.4, Thermo Scientific), allowing up to two missed cleavages and a mass tolerance of 10 ppm for precursor ions and 0.8 Da for the product ions. Oxidation of methionine and carbamidomethylation of cysteine residues, Schiff base and Michael adducts of HNE, PONPC and POVPC on lysine, arginine and cysteine residues were used as variable modifications.

### 2.6 Culturing of cells on collagen

PureCol Type I bovine collagen solution (0.75 mg/ml) was modified by a reaction containing either of 100 μg/ml HNE, 600 μg/ml OxPAPC, 180 μg/ml PONPC or 180 μg/ml POVPC as described above. 300 μl of the mixture obtained was immediately applied per well in a 12-well cell culture plate (Costar, Corning Incorporated, USA) to coat each well, 600 μl per well were used to coat one well of a 6-well tissue culture plate (Costar, Corning Incorporated, USA), and 100 μl were used to coat each chamber of a 4-chamber tissue culture treated glass slide (Falcon, Corning Incorporated, USA). Coating was achieved by incubation of plates/chambers for 24 h at 37 °C. Afterwards, the thin collagen layer was washed two times with PBS and the appropriate cell culture medium to wash out unbound lipid aldehyde species.

Primary fibroblasts from different donors were seeded at a density of 8000 cells/cm^2^ and grown for up to 5 days with regular medium changes as depicted in Supplementary Fig. 1A. For the transwell experiment, fibroblasts were seeded in cell culture inserts with a pore size of 0.4 μm (Thermo Scientific, Denmark) and transferred into a 12-well cell culture plate coated at the bottom with modified collagen for 3 days.

THP-1 cells at a density of 0.5 × 10^6^/ml were treated with 10 nM phorbol-12-myristate-13-acetate (PMA, Sigma-Aldrich, USA) for 24 h to differentiate them into M0 macrophages. Then, the M0 macrophages were detached with accutase (BioLegend, USA) and grown on the previously washed matrix for 24 h (Supplementary Fig. 1A). To investigate the role of free ROS, 5 mM N-Acetylcysteine (NAC, Sigma-Aldrich, USA) was added immediately to the seeded cells. After 24 h of culture, lipopolysaccharide (LPS, Sigma-Aldrich, USA) was added to the cells to a final concentration of 500 ng/ml for 2 h to activate Toll-like receptor 4 (TLR4). For a follow-up experiment, the cells were treated with additional stimuli for 2 h as well. In detail, human TNFα (InvitrogenThermo Fisher, USA) was added to a final concentration of 100 ng/ml, 0.25 μg/ml of Flagellin (TLR5, InvivoGen, France), 0.5 μg/ml of FSL-1 (TLR2, TLR6, InvivoGen) and 20 μg/ml of Poly(I:C) (TLR3, InvivoGen) [31,32]. Afterwards, the RNA was harvested using TRIzol (Invitrogen, Thermo Fisher, USA).

For the cultivation of keratinocytes, collagen type IV was used to mimic the basal lamina. The collagen (Bornstein and Traub Type IV, Sigma-Aldrich, USA) was modified with lipids as described before. Primary keratinocytes from different donors were seeded onto the washed collagen layer with a density of 4500 cells/cm^2^ and incubated for 24 or 72 h with medium change if needed (Supplementary Fig. 1A).

### 2.7 Detection of ROS formation

Intracellular ROS formation was assessed using the CellROX Green Reagent (Invitrogen, Thermo Fisher, USA). Primary fibroblasts grown on modified collagen for 3 days were then incubated with 5 μM CellROX for 30 min at 37 °C. After fixation with formaldehyde (3.7% v/v; SAV Liquid Production GmbH, Germany), cell nuclei were stained with 1 μM Hoechst 33342 (Invitrogen, Thermo Fisher, USA). CellROX exhibits a bright green photostable fluorescence signal upon oxidation by ROS and binding to DNA, with absorption/emission maxima of 485/520 nm, which was examined by confocal microscopy (LSM700, Zeiss). Mean fluorescence intensity was quantified using Fiji (ImageJ 1.54p, Java 1.8.0_322 [64-bit], National Institutes of Health, USA) at 12 sites per field of view per condition.

### 2.8 BODIPY C11

Lipid peroxidation was analyzed using BODIPY 581/591C11 undecanoic acid (Invitrogen, Thermo Fisher, USA). Fibroblasts cultured on a modified matrix were incubated with 2 μM BODIPY C11 and 1 μM Hoechst 33342 in the culture medium for 30 min in darkness at 37 °C. The lipid peroxidation sensor BODIPY C11 localizes to membranes in living cells. Upon oxidation of the polyunsaturated butadienyl portion of the fatty acid analogue in BODIPY C11, the fluorescence emission peak shifts from red ∼590 nm to green ∼510 nm, detectable by confocal microscopy (LSM700, Zeiss). The fluorescent intensity of 5 images per condition separated by the red and green signals was quantified using Fiji (ImageJ, USA) and the ratio of oxidized (green, 485/520 nm) to unoxidized (red, 581/595 nm) signals was calculated using GraphPad Prism (software 8.4.3, USA).

### 2.9 Histology and immunostaining

Human skin obtained from the sun protected upper leg of a 30-year young women was irradiated with 80 J/cm^2^ UVA-1 (Sellamed 3000 device, USA) for 3 weeks to have an accumulated UVA-1 fluence of 600 J/cm^2^ as in Ref. [33]. This would reflect 28 h of unprotected sun exposure at noon at a northern latitude of 35° while 30% would reach the dermis [34]. The UVA irradiated human skin was embedded in Optimal Cutting Temperature (O.C.T.) compound (Scigen Scientific Gardena, USA), snap-frozen in liquid nitrogen, and sectioned at 5 μm thickness. Sections were stained with anti-4-HNE Michael adducts antibody (Calbiochem 393207, 1:100), [35], blocked with normal rabbit serum (Dako, USA) and co-stained with anti-Col1A1 (ab34710, 1:250) which was directly labelled using FlexAble CoraLite Plus 488 antibody labeling kit for rabbit IgG (Proteintech Group, USA).

Fibroblasts cultured on modified collagen for 72 h were fixed with methanol for 20 min at room temperature (RT) and subsequently stained with anti-oxidized phospholipid E06 IgM [36] (Absolute Antibody, Ab02746, 1:100), *anti*-HNE (1:100), and *anti*-Ki67 (Abcam, ab15580, 1:1000). For image analysis, Ki67 positive cells were counted on 5 images per condition and normalized to the total cell count. To investigate the spreading area of fibroblasts cultured on the matrices for 72 h, cells were fixed with 3.7 % formaldehyde and fluorescent phalloidin (Alexa Fluor 568, Invitrogen, USA) was used to stain F-actin. Confocal images acquired at 10x resolution were imported into Strata-Quest (Version 7.1.1.145, Tissue Gnostics) for quantitative analysis. The AF555 channel (phalloidin staining) was smoothed using a Gaussian filter. Nuclei were detected in the DAPI channel and a corresponding cell mask was generated by expanding the nuclear objects within the phalloidin signal. Based on this cell mask, cellular area and perimeter were measured from 5 fields per view per condition.

Paraffin-embedded skin equivalent (SE) sections were stained with hematoxylin and eosin. Cell nuclei in the upper layer of the epidermis, specifically in the stratum corneum, were counted in 10 fields per view and condition.

For immunofluorescence staining, formalin-fixed paraffin embedded sections of the SE were deparaffinized, rehydrated, and antigen retrieval was performed using R-Universal Epitope Recovery Buffer pH 6.0 (Aptum Technologies, Canada). Sections were stained using antibodies against Keratin 10 (BioLegend, PRB-159P, 1:500), Krt14 (Abcam, ab7800, 1:200), LaminB1 (Abcam, ab16048, 1:1000), γ H2AX (Abcam, ab26350, 1:500) followed by Alexa Fluor 546 donkey anti-rabbit (Invitrogen, A10040, Thermo Fisher, USA) or 488 anti-mouse antibody (A21202). Nucleus staining was performed using Hoechst 33342 (Thermo Fisher, USA), and sections were mounted with Dako Fluorescence Mounting Medium (Agilent Technologies, USA). Images were acquired with a Fluorescence Microscope (BX63 Upright Microscope, Olympus) and for higher resolution images a confocal microscope (LSM700, Zeiss) with the software ZEN 2012 SP1 (black edition, version 8.1.0.484) was used. Epidermal thickness of 4 skin equivalents per condition was measured with the ImageJ ‘line’ tool. LaminB1 intensity was measured in a virtual cross section through the epidermal cells, created with the ‘segmented line’ tool. Subsequently the ‘Plot Profile’ of the cross section was generated, the data set was transferred into Microsoft Excel and the mean intensity per pixel was calculated from the sum of positive intensities to the length of the line segment [37]. For analysis of γ H2AX three whole skin sections per condition were recorded using TissueFAXS I PLUS slide-scanning microscope (Zeiss Observer Z1, 12-slide stage, Hamamatsu Orca Flash 4.0 V3 4K, X-Cite Series 120PCQ Laser). Staining intensity per cell was measured using Strata-Quest image analysis software (version 5.0.1.336). The γ H2AX positive cells were selected using the software built-in gating capability. Final data analysis was performed in MATLAB R2024a (MathWorks).

### 2.10 RNA extraction and quantitative RT-PCR analysis

Total RNA from cells was isolated using TRIzol Reagent (Invitrogen, Thermo Fisher, USA) and further processed following manufacturer's instructions. Reverse transcription into cDNA was performed with the iScript cDNA Synthesis Kit (Bio-Rad Laboratories, Germany) according to supplier's protocol for 1 μg total RNA template per reaction. Quantitative real-time polymerase chain reaction (RT-PCR) analysis was conducted on a thermal cycler (CFX96 Real -Time System, Bio-Rad) using LightCycler 480 SYBR Green I Master mix (Roche, Switzerland) as described previously [38]. Primer sequences are listed in the Supplementary Table 1. The mathematical model of Pfaffl [39] was used for relative quantification and expression of the target gene was normalized to the housekeeping gene β-2-microglobulin (B2M). Robustness of the normalization strategy was confirmed by obtaining identical relative expression patterns when eukaryotic elongation factor 1-alpha 1(EEF1A1) was used as an alternative reference gene in macrophages.

### 2.11 Full thickness human skin equivalents

In vitro reconstructed skin equivalents (SE) were generated as described previously [40]. To obtain modified collagen, HNE or OxPAPC were dissolved at subsequent concentrations 2.5 μg/ml or 25 μg/ml in K_2_HPO_4_ buffer supplemented with 1% d-glucose (Sigma-Aldrich, USA), 0.5 % phenol red solution (Sigma-Aldrich, USA) and diluted one to ten using DPBS (10X). PureCol Type I bovine collagen solution (3.1 mg/ml, Advanced BioMatrix, Sweden) was added and incubated for 4 h at 37 °C to modify 1/4th of the collagen. Then, the remaining modified collagen solution was added together with 10% fibroblasts in FBS (∼250 000 FB per SE). The mixture, adjusted to a ∼ pH 7.4 with 1 M NaOH, was poured into 3.0 μm pore size Cell Culture Inserts (Falcon, Corning, USA), which were placed in deep six-well trays (Corning, USA). After gelation at 37 °C in a humidified atmosphere for 3 h in the absence of CO_2_, gels were equilibrated in Keratinocyte Growth Medium (KGM2, PromoCell, Germany) and placed into an incubator with 5% CO_2_. The next day, keratinocytes were seeded on top of the dermal equivalents (∼400 000 KC per SE). After detaching the skin equivalents, they were grown at the air-liquid interface for 5 days using KGM2 without bovine pituitary extract (BPE) and epinephrin but supplemented with 0.1% BSA (w/v, Sigma-Aldrich, USA), 0.05 mg/ml l-ascorbic acid (Sigma-Aldrich, USA) and 1 M CaCl_2_ (Sigma-Aldrich, USA). Histology samples were taken by punch biopsy, formalin-fixed or snap frozen in O.C.T. The remaining skin equivalents were divided into epidermal and dermal fractions using tweezers and harvested in TRIzol or protein lysis buffer for further analyses.

### 2.12 Cytokine array

To determine cytokine protein levels, supernatants of the biological quadruplicates of THP-1 cells cultured on the modified matrix were pooled and subjected to a Proteome Profiler Human XL Cytokine Array (R&D Systems, USA) according to the manufacturer's instructions. Signals were developed on a ChemiDoc XRS + device (Bio-Rad, USA). The dot intensities were calculated for each cytokine duplicate using the volume tool implicated in ImageLab 6.1 (Bio-Rad). Mean background corrected dot signal intensities were presented in bar chart. Significant differences between samples were determined by GraphPad Prism and visualized as red or blue boxes around the respective cytokines.

### 2.13 Western blotting analysis

Proteins were harvested using the 4x Laemmli Sample Lysis Buffer (Bio-Rad) containing protease and phosphatase inhibitors. Criterion electrophoresis system (Bio-Rad) was used to perform an SDS-PAGE (4%–12 %, Bio-Rad) run at 200 V for 1 h to ensure collagen separation or 30 min for cell lysates. The proteins were blotted onto a nitrocellulose membrane (Bio-Rad) using the Trans-Blot® Turbo Transfer System (Bio-Rad) and blocked with 5 % dry milk (Sigma-Aldrich, USA) in PBS containing 0.1 % Tween 20 (Bio-Rad). The membranes were then incubated with *anti*-P16 (Abcam, ab108349, 1:1000) or *anti*-β-Tubulin (Abcam, ab6046, 1:5000) overnight at 4 °C, followed by incubation with the corresponding secondary antibody, anti-rabbit IgG-HRP (Bio-Rad 1706515, 1:10000) for 1 h at RT. After washing, the blots were developed using the SuperSignal West Dura Extended Duration Substrate (Thermo Fisher, USA). Labelled bands were quantified using ImageLab 6.1 (Bio-Rad) using the mean intensity and normalized to the tubulin signal.

### 2.14 Statistical analysis

All statistical analyses and graphical presentation of the data were performed using GraphPad Prism software (version 8.4.3, USA). Significant outliers were determined using Grubbs' test with alpha = 0.05 (Outlier Calculator, GraphPad) and removed only when statistically supported by this test. Additionally, qPCR values were excluded when amplification failed (no Ct detected) or when melting curves showed aberrant or non-specific peaks. For all qPCR experiments, we performed biological quadruplicates (n = 4) using multiple independent donors, as shown in the figures and indicated in the respective figure legends. For experiments involving the THP-1 cell line, an additional technical replicate experiment was included. All staining experiments were conducted using at least two independent donors. Skin equivalent experiments were performed three independent times using different donor combinations and each experiment included biological duplicates (n = 6). Statistical significance was determined by Ordinary one-way ANOVA, two-way ANOVA or Welch's *t*-Test respectively Unpaired *t*-test both with prior F test to compare variances. For qPCR, the variance homogeneity was evaluated by Brown-Forsythe and Bartlett's test followed by a Dunnet's or Turkey's multiple comparison test. The significance of differences was indicated in the figures by asterisk and if not stated otherwise represented by the following *p < 0.05, **p < 0.01, ***p < 0.005, ****p < 0.001.

## 3 Results

### 3.1 Collagen is covalently modified by senescence associated, reactive aldehydic lipids and lipid peroxidation products

**Fig. 1:**
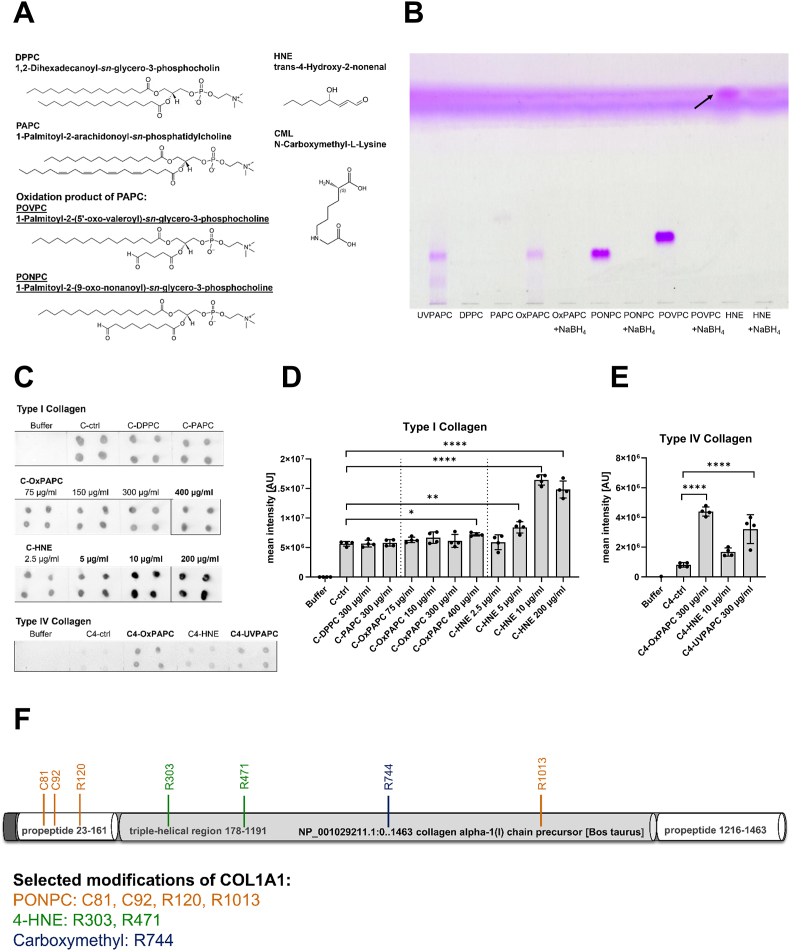
Collagen types I, II, and IV are covalently modified by senescence-associated reactive aldehydic lipids and lipid peroxidation products. **A** Chemical structures of the lipid species used in this study. Underlined names indicate lipids previously identified as components of the SASP and reported to be elevated in senescent fibroblasts. **B** Schiff's reagent detects aldehydic groups in oxidized lipids (10 μg each applied to TLC), which are reducible with NaBH_4_. The black arrow indicates HNE migrating to the top of the plate due to its distinct polarity. **C** OxyBlot analysis using DNPH detects aldehydes in lipids covalently bound to collagen types I and IV, applied as dots on a nitrocellulose membrane. Significant differences to the corresponding control are indicated by bold caption. **D** Bar chart of collagen type I and **E** collagen type IV signal intensity of the dots showing mean ± SD, n = 4. Asterisks represent significant differences (*p < 0.05; **p < 0.01; ***p < 0.005; ****p < 0.001) determined by one-way ANOVA. **F** Selected high and low molecular weight modifications to specific amino acid residues of COL1A1 identified with LC-MS/MS.

Cells reaching senescence accumulate and secrete oxidized lipid species [41,42]. To investigate whether potentially reactive lipid aldehyde species present in the SASP of fibroblasts [12] and melanocytes [9] would be able to adduct to collagen, we first determined their ability to form Schiff bases under controlled reaction conditions. To this end we tested 1-palmitoyl-2-(5′-oxo-valeroyl)-*sn*-glycero-3-phosphocholine (POVPC) and 1-palmitoyl-2-(9′-oxo-nonanoyl)-*sn*-glycero-3-phosphocholine (PONPC). These aldehydic phospholipids were found to be part of the secretory phenotype and are oxidation products of 1-palmitoyl-2-arachidonoyl-*sn*-phosphatidylcholine (PAPC) and 1-palmitoyl-2-linoleoyl-*sn*-glycero-3-phosphocholine (PLPC), respectively (Fig. 1A). Further, we tested PAPC that had been prior subjected to autoxidation in ambient air (OxPAPC) or to UVA photooxidation (UVPAPC), both yielding a complex mixture of oxidation products, which contain POVPC [43,44]. Moreover, we tested 4-hydroxynonenal (HNE), an intensively studied reference aldehydic lipid peroxidation product (LPO). We used the saturated phospholipid 1,2-Dipalmitoyl-*sn*-glycero-3-phosphocholine (DPPC) which is structurally similar to PAPC or PLPC as a non-oxidizable control. We separated the lipids using thin layer chromatography (TLC), stained the plates with the Schiff's reagent, and confirmed presence of aldehydes by Schiff base formation in PONPC, POVPC, HNE, and to a lesser extent in UV or air oxidized forms of PAPC as evidenced by the color reaction (Fig. 1B). We corroborated the results as the Schiff base reaction was lost upon prior reduction of the carbonyl groups with NaBH_4_ (lanes 5, 7, 9, and 11).

Next, we investigated the ability of the lipid aldehydes to react with bovine collagen type I and II and with human collagen IV, which are common collagen isolates used in the cell culture. We incubated the lipids with the collagens according to a lipid adduction protocol [26] and then spotted the samples onto a nitrocellulose membrane. Then we assayed protein carbonyl formation with dinitrophenylhydrazine (DNPH), which reacts with aldehydes or ketones to form hydrazones that are specifically detectable with an *anti*-DNP antibody (Fig. 1C). HNE adduction to collagen (C-HNE) resulted in a concentration-dependent increase in DNP signal intensity (Fig. 1D), which was significantly higher compared to unmodified collagen controls (C-ctrl). When collagen was incubated with OxPAPC (C-OxPAPC), we detected significant formation of the DNP signal only with the highest concentration of 400 μg/ml. This aligns with the previous findings that only a small fraction of the oxidation products present in OxPAPC are aldehydes [45]. Lipids lacking reactive carbonyl groups, such as C-DPPC and C-PAPC, exhibited signal intensities comparable to the control reaction without lipids, indicating no collagen modification. Compared to bovine collagen type I, both HNE and OxPAPC induced more pronounced modifications in human collagen type IV, which also exhibited reduced background signal (Fig. 1E). Further, we performed a mass spectrometric analysis of bovine collagen type I and II exposed to oxidized PAPC prepared either by autoxidation (OxPAPC) or photooxidation (UVPAPC). Liquid chromatography-tandem mass spectrometry (LC-MS/MS) identified protein modifications by oxidized lipids already present in the collagen preparation and specific adducts that were introduced by the exposure to OxPAPC (Fig. 1F). Among high molecular weight (HMW) modifications were Schiff base adducts of PONPC on cysteine (C81, C92) and arginine (R120, R1013) residues of COL1A1. Apart from that, truncated lipid peroxidation products, including Michael adducts of HNE on arginine (R302, R471) of collagen alpha-1(I) chain, were identified. Also, carboxymethyl modifications formed by glyoxal on COL1A1 (R744) were detected. A comprehensive list of all the specific modifications to COL1A1, COL1A2, COL2A1, and COL3A1 is provided in the Supplementary Table 2. Another example of LMW modifications were MDA adducts (methylglyoxal-derived hydroimidazolone) on R438 of collagen alpha-2(I) chain. Summary of the statistical analysis revealed that mainly arginine, followed by lysine and to a small extent histidine and cysteine were modified by HMW lipid peroxidation products.

### 3.2 Matrix changes can be induced by oxidized lipids *in vitro* and *in vivo*

**Fig. 2:**
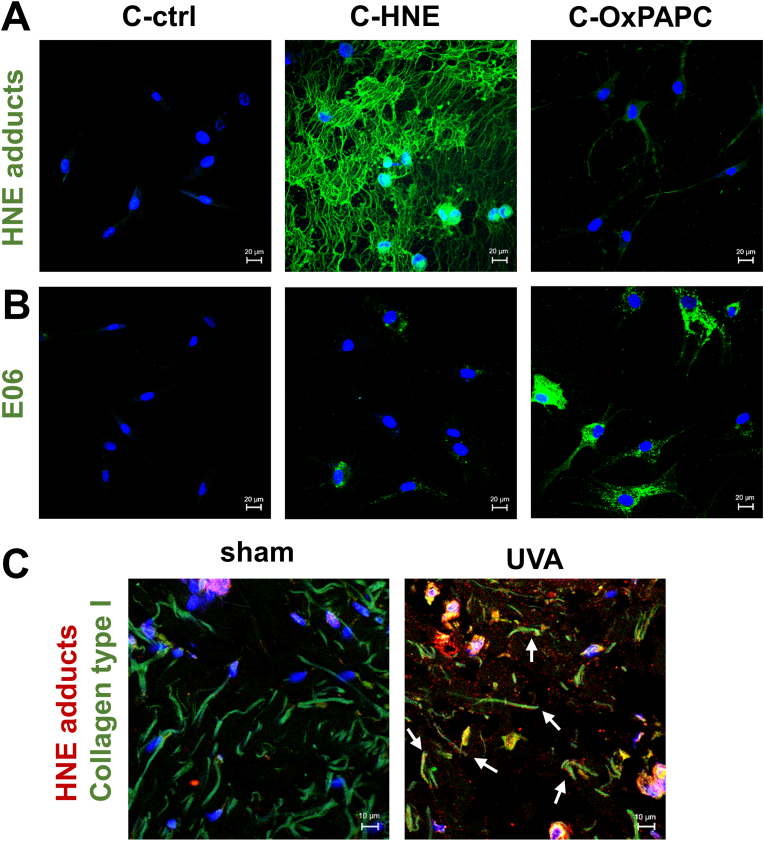
Oxidized lipids induce matrix changes *in vitro* and *in vivo*. **A** Representative images of fibroblasts (f37y, f42y) cultured on collagen modified *in vitro* with HNE and OxPAPC. Nuclei were stained with Hoechst, and matrix modifications were detected using **A***anti*-HNE Michael adducts antibody and **B***anti*-E06 antibody, respectively. **C** Confocal images of human skin (f30y), irradiated for three weeks with a cumulative dose of 600 J/cm^2^ UVA. White arrows point to colocalization (yellow) of anti-collagen type I (green) and *anti*-HNE Michael adducts antibody signal (red), indicating modified collagen fibers in the immunostained tissue. Scale bars: A, B = 20 μm; C = 10 μm.

To investigate how fibroblasts, the main cells of the dermis, would be influenced by lipid modified collagen, we conducted a series of immunohistochemical and biochemical analyses on cells cultured on the matrices for 3 days. To that end, we coated cell culture plates with the lipid-modified collagen and removed unbound lipids by repeated washes before seeding primary fibroblasts. After three days of culture, we stained the cells with antibodies that can detect oxidized moieties of lipids. Immunostaining with *anti*-HNE Michael adducts antibody revealed a dense network of *in vitro* HNE-modified collagen (Fig. 2A) also in the cell free control experiment (Supplementary Fig. 1B). In contrast, staining with E06 antibody, which recognizes the phosphatidylcholine headgroup of oxidized phospholipids as an epitope [46] revealed a cell-associated signal in fibroblasts grown on C-OxPAPC comparable to the pattern observed in UVA irradiated fibroblasts [47] (Fig. 2B). Notably, cell free staining of the OxPAPC modified collagen both with *anti*-HNE and *anti*-E06 revealed unspecific immunoreactivity. This pattern may be explained by either uptake and incorporation of oxidized epitopes and/or by induction of a lipid peroxidation reaction inside the cells.

To investigate whether the collagen modification could be detected also in human skin, biopsies obtained from a young donor were irradiated with a total dose of 600 J/cm^2^ UVA to induce HNE formation [33]. Immunostaining with antibodies against collagen type I and HNE Michael adducts revealed co-localization of the signals (Fig. 2C, Supplementary Fig. 1C). Also, in contrast to the sham control, the immunostained collagen fibers appeared shorter and fragmented fitting to the elevated MMP1 protein content of the dermis (Supplementary Fig. 1D)

### 3.3 Growth on modified collagen reduces cell proliferation, changes spreading area and elicits ROS generation

**Fig. 3:**
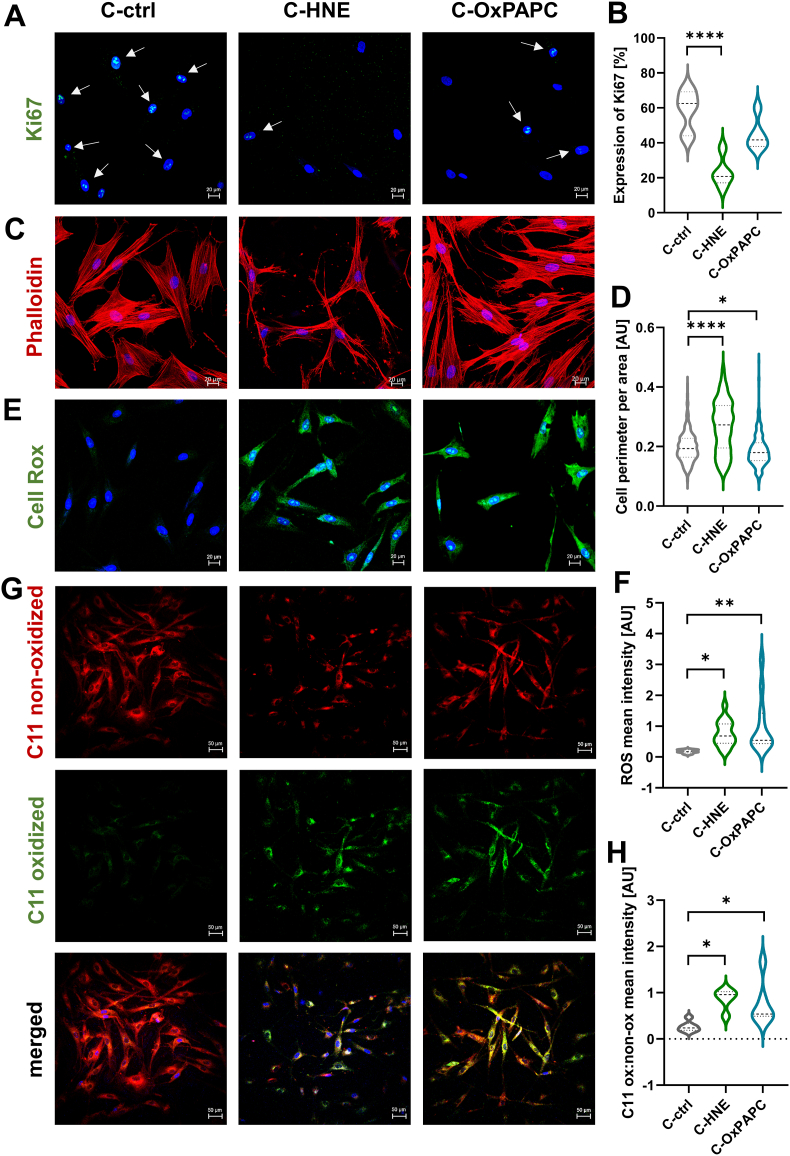
Growth on modified collagen reduces cell proliferation, changes spreading area and elicits ROS generation. **A** Confocal images of *anti*-Ki67 immunostaining of fibroblasts (f37y, f42y) cultured on modified collagen for 72 h. White arrows indicate Ki67 positive cells. **B** Violin plot showing the quantification of cells expressing Ki67 normalized to the total cell count in percent, n = 5. **C** Phalloidin staining of fibroblasts (f33y) cultured on modified collagen for 72 h. **D** Quantification of cell perimeter normalized to cell area using StrataQuest tissue cytometry software, n = 5. **E** Representative images of primary fibroblasts (f37y, f42y) stained with CellROX after 3 days growth on modified collagen to determine ROS levels. **F** Violin plot showing mean CellROX signal intensity, n = 12. **G** Representative images of fibroblasts (f37y, f42y) incubated with Bodipy C11 after 72 h on modified matrix. Merged and split channels depict oxidized (green) and non-oxidized (red) signals **H** Violin plot showing the oxidized to non-oxidized signal ratio, n = 5. Nuclei were counterstained with Hoechst. Violin plots depict data distribution and density, with the median and interquartile range indicated. Statistical significance was determined by one-way ANOVA and is represented by asterisks (*p < 0.05; **p < 0.01; ***p < 0.005; ****p < 0.001). Scale bars A, C, E = 20 μm; G = 50 μm.

To investigate how fibroblasts react to a microenvironment of modified collagen, we cultured cells for three days on modified collagen and stained for the cell proliferation marker Ki67 (Fig. 3A). We observed a significantly lower number of Ki67 positive nuclei in FB grown on C-HNE (Fig. 3B).

To assess whether the observed changes would be driven by altered receptor interactions, we performed Phalloidin staining on these cells. The fluorescent dye binds to F-actin which allowed to analyze the area of spreading. Fibroblasts cultured on HNE modified collagen appeared thin and long with more dendrites while C-OxPAPC led to less prominent changes (Fig. 3C). Analysis of the cell perimeter normalized to the whole cell area revealed significantly higher values for fibroblasts on C-HNE corroborating the stretched appearance (Fig. 3D, quantification details in Supplementary Fig. 2A). Since actin filaments and integrins govern cell attachment to collagen [48], we examined the genes encoding for Integrin α1, α2, and α11 (Supplementary Fig. 2B). The expression of ITGA1 differed significantly between fibroblasts grown on C-ctrl and C-HNE indicating altered collagen interaction [49].

Moreover, we assessed reactive oxygen species (ROS) levels using the CellROX reagent which binds to DNA upon oxidation. We detected significant accumulation of the fluorogenic probe in the nucleus and mitochondria as well as in the cytoplasm of fibroblasts cultured on C-HNE and C-OxPAPC (Fig. 3E and F).

Next, to investigate whether culturing on modified matrices promotes lipoxidation, we measured the ratio of unoxidized to oxidized lipids using BODIPY C11. This live-cell lipid peroxidation sensor shifts from red to green fluorescence emission upon oxidation of the polyunsaturated butadienyl portion of its fatty acid analog. We found that fibroblasts grown on unmodified collagen (C-ctrl) predominantly emitted red/non-oxidized (∼590 nm) signal (Fig. 3G). In contrast, culture on both of the modified matrices resulted in a shift of the fluorescence emission towards green (∼510 nm), suggesting a significantly higher ratio of oxidized to non-oxidized lipids on C-HNE and C-OxPAPC (Fig. 3H) supporting the initiation of lipid oxidation.

### 3.4 Fibroblasts cultured *in vitro* on modified collagen I display inflammatory and stress responses

**Fig. 4:**
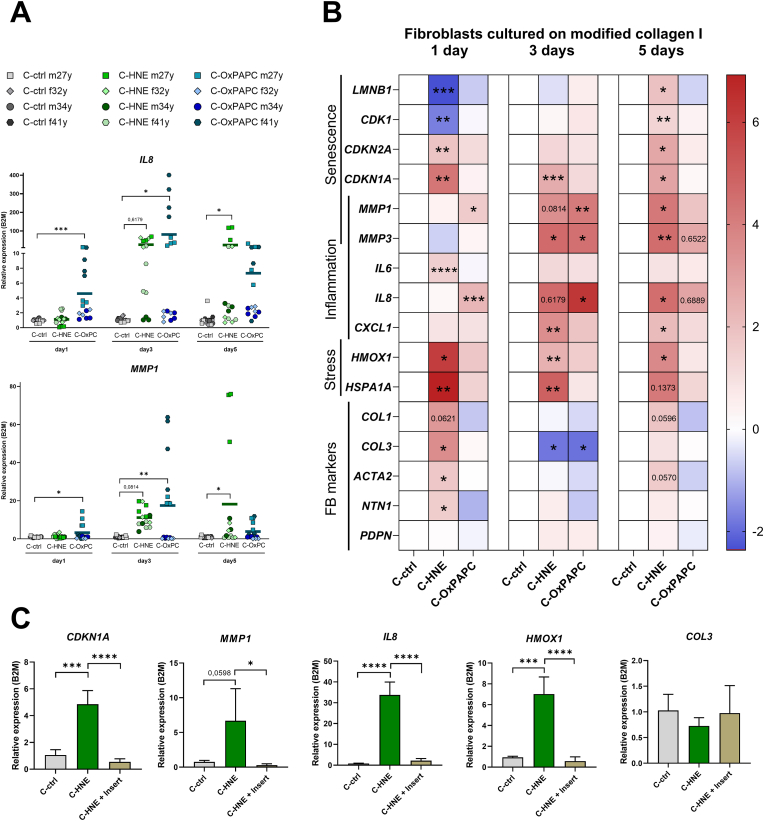
Fibroblasts cultured on *in vitro* modified collagen type I exhibit inflammatory and stress responses. **A** Scatter plots show gene expression levels of *IL8* and *MMP1* relative to *B2M* in fibroblasts cultured on HNE or OxPAPC modified collagen. Primary fibroblasts from four donors (m27y, f34y, m32y, f41y) were grown in quadruplicates for up to five days, n = 16. **B** Heatmap of log_2_-transformed relative expression levels normalized to the corresponding control. Transcriptional levels of senescence, inflammation, stress, and fibroblast morphology markers were assessed. Asterisks indicate significant differences, with p-values shown for a z value > 1. Colors represent expression changes (blue = downregulation, red = upregulation). **C** Bar charts showing relative expression levels of fibroblasts (f23y) with direct contact to HNE modified collagen (C-HNE) or without direct contact (C-HNE + Insert), mean ± SD, n = 4. Statistical significance of the mean was determined by one-way ANOVA and is indicated by asterisks (*p < 0.05; **p < 0.01; ***p < 0.005; ****p < 0.001).

Next, we analyzed gene expression in fibroblasts grown on modified collagen. We cultured primary fibroblasts from four different donors for 24 h, 72 h, and up to 120 h to record immediate and long-term effects on the cell fate. We used PCR-based gene expression panels to investigate markers of senescence (*LMNB1, CDK1, CDKN2A, CDKN1A, MMP1, MMP3*), inflammation (*IL6, IL8, CXCL1, MMP1, MMP*), electrophilic stress response (*HMOX1, HSPA1A*) alongside with general fibroblast markers (*COL1, COL3, ACTA2, NTN1, PDPN*). In detail, we observed significantly higher levels of *IL8* and *MMP1* expression in cells grown on OxPAPC modified collagen (Fig. 4A). After five days, the genes were also significantly upregulated with C-HNE.

We analyzed relative expression levels of 16 genes (Supplementary Fig. 3A and B) and summarized the data in a heatmap (Fig. 4B) to highlight differentially expressed genes over the five-day time course. We next investigated whether contact to matrix modified with senescence associated lipids would itself promote senescence. HNE modification resulted in an early senescence signature from day 1. In detail, we found that *LMNB1* and *CDK1* were transiently lower expressed while cell cycle inhibitor expression of *CDKN2A* encoding for P16 and *CDKN1A* encoding for P21 was upregulated during the whole time in culture on HNE modified matrix.

Culturing fibroblasts on OxPAPC modified matrix resulted in transiently significantly higher levels of *MMP1* and *MMP3* while collagen type III (*COL3*) expression was reduced. Expression of matrix degrading enzymes (*MMP1, MMP3*) was elevated in fibroblast grown on C-HNE. Overall, both OxPAPC and HNE modifications triggered a delayed matrix remodeling response.

Furthermore, OxPAPC modification resulted in an early electrophilic response from day 1-3 while C-HNE promoted an early cytokine expression. Moreover, HNE modified matrix led to a significantly higher stress response over an extended time course shown by *HMOX1* and an unfolded protein response (UPR) displayed by *HSPA1A* expression.

To assess whether fibroblasts adopted a more papillary phenotype (*PDPN, NTN1*) or acquired myofibroblast features (*ACTA2*), we analyzed their expression patterns. One day after culturing on C-HNE, we observed significantly higher levels of *NTN1* and *ACTA2* encoding for αSMA, while *PDPN* expression was unchanged. Together, these data suggest that C-HNE exposure promotes a papillary-like, activated fibroblast morphology. The spindle-shaped morphology was also evident in Phalloidin staining (Fig. 3B).

To confirm that these effects are due to the modified collagen matrix rather than free bioactive lipids, we performed a transwell experiment in which fibroblasts were cultured in inserts without direct contact to the HNE-modified collagen. Corroborating the results from experiments shown in Fig. 4B, HNE modified collagen results in significant higher expression of *CDKN1A, MMP1, IL8* and *HMOX1* and lower expression of *COL3* (Fig. 4C). Importantly, fibroblasts cultured in the insert showed the same expression pattern as the unmodified collagen control as there was no direct contact to the matrix.

### 3.5 Macrophages cultured on modified collagen I show early senescence markers, low grade inflammation, and an impaired response to LPS

**Fig. 5:**
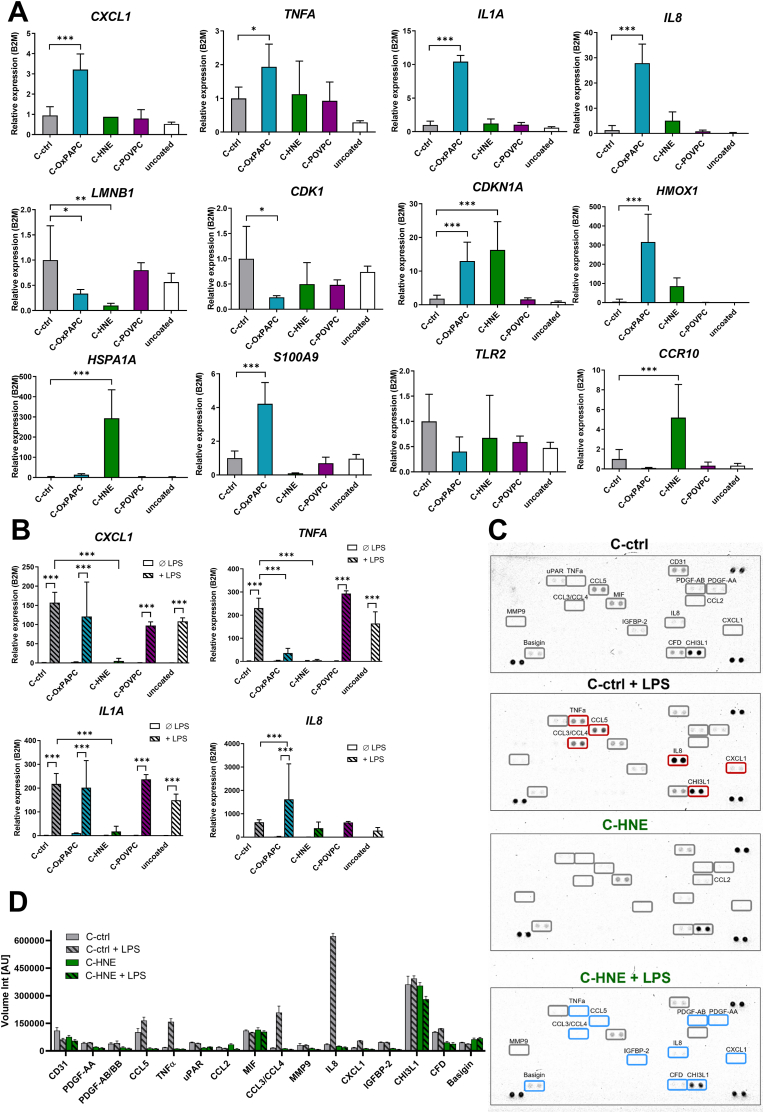
Macrophages cultured on modified collagen type I show early senescence and inflammatory markers but impaired response to LPS. **A** Bar charts with solid-colored bars represent the effect of the modified matrix on macrophages. THP-1 cells were treated with 10 nM PMA and cultured on modified collagen for 24 h. Expression levels of cytokines/chemokines (*CXCL1, TNFA, IL1A, IL8*), senescence markers (*LMNB1, CDK1, CDKN1A*), stress response genes (*HMOX1, HSPA1A*), and receptor genes (*TLR2, CCR10*) were analyzed relative to *B2M*, mean ± SD, n = 4. **B** Bar charts with dotted bars show the effect after the LPS stimulation (TLR4 agonist). Cells were treated with 500 ng/ml LPS for 2 h, n = 4. Asterisks indicate statistical significance (*p < 0.05; **p < 0.01; ***p < 0.005) determined by one-way ANOVA. **C** Cytokine array membranes incubated with pooled supernatants, n = 4 of the macrophages grown on collagen and HNE modified collagen, both without and with LPS stimulus. Grey boxes with labels mark detected cytokines. Red boxes highlight cytokines significantly upregulated upon LPS stimulation of macrophages cultured on collagen (C-ctrl + LPS) compared to untreated control (C-ctrl). Blue boxes indicate cytokines significantly downregulated upon LPS in C-HNE compared to C-ctrl + LPS. **D** Bar chart showing colorimetric quantification of the dots on the respective membranes, mean ± SD, n = 2.

The SASP of fibroblasts can influence the behavior of monocyte derived cells in the dermis [23]. To investigate whether culture on a SASP-lipid modified matrix would be sufficient to affect the mRNA expression profile of cytokine genes in monocytes, we performed analysis of steady state mRNA expression. We differentiated THP-1 cells with PMA to M0 macrophages and seeded them on plates pre-coated with a modified collagen type I matrix, which were washed prior cell seeding to remove unbound lipid species. We observed significant upregulation of *CXCL1*, S100A9, *HMOX1*, *IL1A*, *IL8* and *TNFA,* after 24 h culture on C-OxPAPC (Fig. 5A) as compared to the control matrix. In contrast, cells grown on HNE modified collagen expressed significantly more *CCR10* and *HSPA1A*. A shared response across both matrices was the significantly reduced expression of *LMNB1* and *CDK1*, along with a marked increase in *CDKN1A* (P21) expression. These findings suggest that while both matrices elicit an “early senescence” transcriptomic signature in macrophages, HNE modification seems to cause an unfolded protein response while OxPAPC modification results in a gene expression pattern consistent with low grade inflammation and electrophilic stress [50,51].

Free oxidized phospholipids can impair Toll-like receptor (TLR) signaling by interfering with the interaction between TLR4, Lipopolysaccharide-binding protein (LBP) and CD14 [44,52,53]. Also, it was shown that the free lipid peroxidation product HNE mediates inflammation by the TLR4/NF-κB-dependent pathway [54]. To investigate whether the function of TLR4 mediated signaling would be affected by contact of the macrophages to the lipid modified collagen, we stimulated the cells with LPS for 2 h before RNA extraction (Fig. 5B). Macrophages cultured on C-ctrl expressed significantly and massively higher levels of *CXCL1, IL1A* and *TNFA* as result of LPS stimulation. We found that macrophages grown on C-HNE had a massively impaired cyto-/chemokine gene expression response upon stimulation with LPS. In macrophages grown on C-OxPAPC we observed on the one hand a reduced inducibility of *TNFA* expression, but on the other hand an increase of LPS-induced *IL8* expression compared to C-ctrl. We did not find any differences in gene expression response to LPS-treatment between macrophages grown on either C-POVPC or uncoated tissue culture plates compared to C-ctrl. Of note, the senescence markers were not influenced by the LPS stimulus (Supplementary Fig. 4).

In general, the normalization strategy proved robust, as identical relative expression patterns were obtained when eukaryotic elongation factor 1-alpha 1 (*EEF1A1*) was used as an alternative reference gene (Supplementary Fig. 5).

To further validate the observed gene expression changes on the protein level, we performed a cytokine array assay (Fig. 5C). The provided membranes were incubated with the supernatants of the monocytes grown on the respective matrices. The panel includes 105 cytokines and dots with a prominent signal were marked with grey boxes. We analyzed the signal intensities (Fig. 5D) and determined significant differences. The cytokines levels of TNF α, CCL3/4, CCL5, IL8, CXCL1 and CHI3L1 were significantly elevated in cells grown on the collagen control matrix with the additional LPS stimulus. Macrophages cultured on HNE modified collagen which received the LPS treatment secreted significantly lower levels of TNF α, CCL3/4, CCL5, PDGF-AB, PDGF-AA, IGFBP-2, IL8, CXCL1, Basigin, CFD and CHI3L1 compared to those on the control matrix. These responses were in accordance with our findings on gene expression.

### 3.6 Free reactive oxygen species affect basal and LPS induced gene expression on lipid modified matrices

**Fig. 6:**
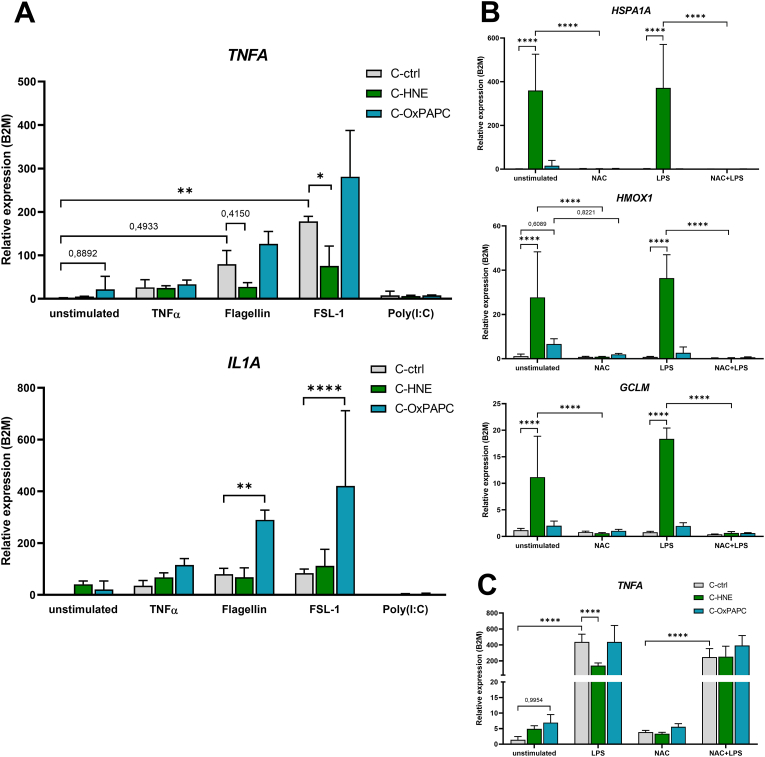
Free reactive oxygen species affect basal and LPS induced gene expression on lipid modified matrices. **A** Bar charts represent relative gene expression of *TNFA* and *IL1A* normalized to *B2M* showing mean ± SD, n = 4. THP-1 cells were activated with 15 nM PMA, cultured on modified matrices for 24 h, and then stimulated for 2 h with human TNFα (100 ng/ml), Flagellin (0.25 μg/ml, TLR5), FSL-1 (0.5 μg/ml, TLR2, TLR6), and Poly(I:C) (20 μg/ml TLR3), respectively. **B, C** Bar charts show relative expression levels of macrophages treated with 5 mM N-Acetylcysteine (NAC) immediately after seeding and cultured for 24 h, followed by stimulation with 500 ng/ml LPS for 2 h, mean ± SD, n = 4. Asterisks indicate statistical significance (*p < 0.05; **p < 0.01; ***p < 0.005; ****p < 0.001), and exact p-values are shown for borderline significance determined by one-way ANOVA.

To assess whether also other pattern recognition receptors and other inflammatory stimuli would be sensitive to matrix modification, we stimulated the macrophages with an agonist panel of Flagellin (TLR5), Fibroblast-stimulating Lipopeptide-1 (FSL-1; TLR2, TLR6), and Polyinosinic-polycytidylic acid (Poly(I:C); TLR3) as well as *TNFα*. The HNE modified collagen resulted also in an impaired response to Flagellin and FSL-1 regarding the induced *TNFA* expression (Fig. 6A).

Interestingly, TLR5 and TLR2/6 induced *IL1A* expression was not impaired by the modified matrix, indicating that for those PRR the agonist activation is still active and that there must be another mechanism that interferes with TLR2/6, 4 and 5 mediated *TNFA* induction. TNF*α* induced expression of *TNFA* mRNA was not affected by the matrix modification, and TLR3 agonism did not induce *TNFA* expression.

Next, we examined whether the generated ROS upon contact with the modified matrix would affect gene expression and the response to LPS. Immediately after seeding THP-1 cells activated with PMA to the native and modified collagen matrices, we added 5 mM of N-Acetylcysteine (NAC). The (Nrf2 dependent) antioxidant stress responses elicited on C-HNE and, at a lesser extent on C-OxPAPC were blunted by NAC, as shown by *HMOX1* and *GCLM* expression (Fig. 6B). Also, C-HNE and again less prominent, C-OxPAPC induced expression of *HSPA1A*, a key marker for the unfolded protein response (UPR), was completely blunted upon NAC treatment. When investigating the LPS induced regulation of *TNFA* expression in this setting, we observed that the C-HNE mediated impairment of LPS induced *TNFA* was restored to the fold change observed on unmodified collagen (Fig. 6C).

Together, these findings suggest that reactive radicals which can be scavenged by NAC act as messengers that mediate stress response gene expression and PRR stimuli observed in cells grown on modified matrix.

### 3.7 Keratinocytes cultured on modified collagen IV show oxidative stress and lower MMP levels

**Fig. 7:**
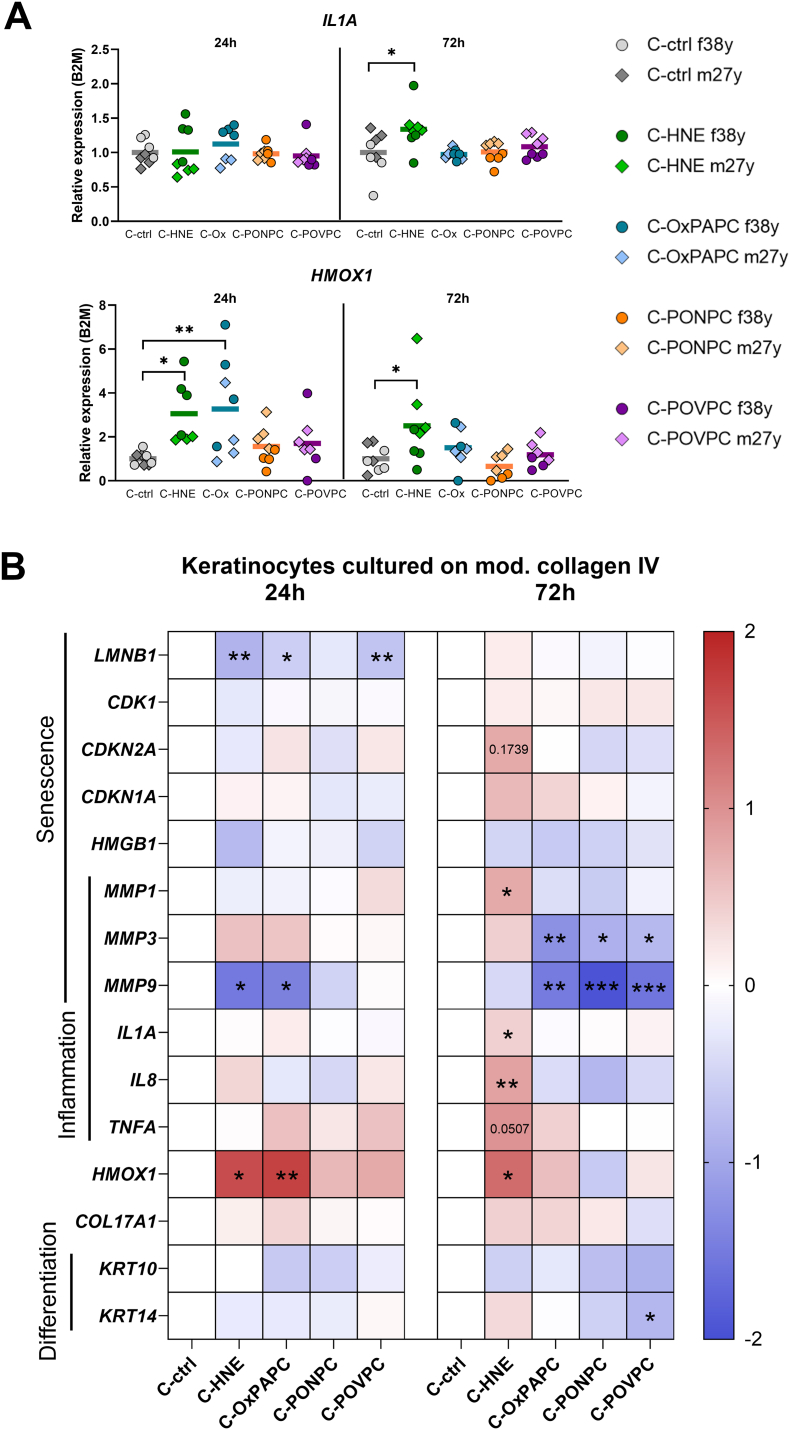
Keratinocytes cultured on modified collagen type IV show oxidative stress and reduced MMP levels. **A** Scatter plots show gene expression levels of *IL1A* and *HMOX1* relative to *B2M* in keratinocytes cultured on HNE or OxPAPC modified collagen type IV. Primary keratinocytes from two donors (f38y, m27y) were grown in quadruplicates for up to 72 h, n = 8. **B** Heatmap of log_2_-transformed relative expression levels normalized to the corresponding control. Transcriptional levels of senescence, inflammation, and keratinocyte differentiation markers were assessed. Colors represent expression changes (blue = downregulation, red = upregulation). Statistical significance was determined by one-way ANOVA and is indicated by asterisks (*p < 0.05; **p < 0.01; ***p < 0.005; ****p < 0.001) with p-values shown for z values > 1.

To investigate functional changes in keratinocytes (KC) exposed to a modified microenvironment, we analyzed gene expression under these conditions. Undifferentiated KCs with proliferative potential are located in the basal layer of the epidermis, where collagen type IV is the dominant basement membrane protein. Therefore, we modified collagen IV and cultured KC on native or modified collagen IV for up to 3 days. We observed that both C-HNE and C-OxPAPC increased oxidative stress response marked by significantly higher *HMOX1* levels. Growth of keratinocytes on HNE modified collagen resulted in significantly higher *IL1A* expression (Fig. 7A).

To gain a broader view of KC responses we generated a relative gene expression heatmap highlighting the regulation of key genes (Fig. 7B, detailed graphs in Supplementary Fig. 6A and B). In summary, we found a trend of senescence marker expression (lower *CDK1*, higher *CDKN2A, CDKN1A* after 72 h), while only *LMNB1* was significantly reduced in HNE, OxPAPC and POVPC modified collagen.

Further, we assessed the matrix remodeling response and found significantly higher *MMP1* levels in cells grown on C-HNE than in controls after 72 h. In contrast, expression of *MMP3* and *MMP9* responsible for degradation of collagen type IV was significantly reduced after 3 days of growth on C-OxPAPC, C-PONPC and C-POVPC. Interestingly, we observed elevated *COL17A1* expression which is responsible to anchor KC to the basal lamina [55], among other functions.

Looking into inflammation markers, growth on HNE modified collagen for 72 h caused an inflammatory phenotype with significant higher expression of *IL1A, IL8* and borderline significant *TNFA* levels.

Moreover, we examined KC differentiation makers and found lower levels of *KRT14* in KC, especially when grown on C-POVPC (Fig. 7B). We speculate that culture on basal lamina collagen IV modulates baseline expression of *COL17A1, KRT10,* and *KRT14*.

### 3.8 Skin equivalents with modified collagen I show disturbed differentiation and an early senescent phenotype of FB and KC

**Fig. 8:**
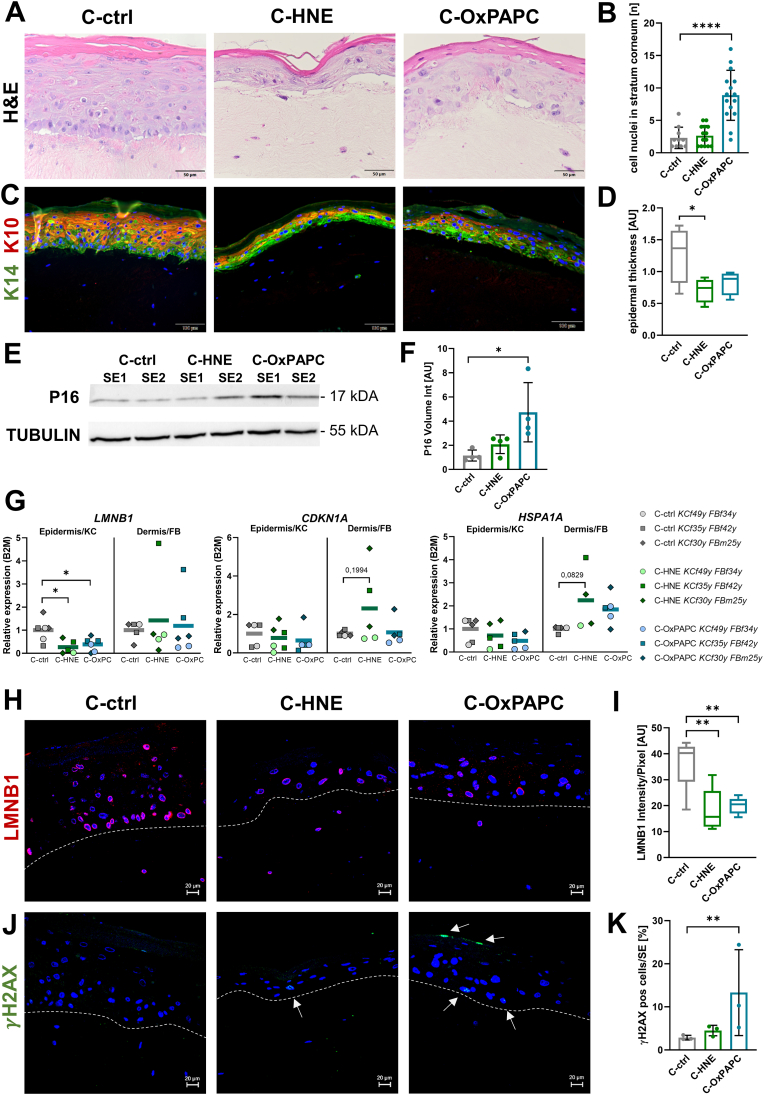
Skin equivalents with modified collagen show disturbed differentiation and early senescence. **A** Hematoxylin & Eosin staining of skin equivalents. Collagen was modified, fibroblasts from different donors (f34y, f42y, m25y) were seeded into the matrix, and keratinocytes (f49y, f35y, f30y) on top; n = 6. SE were cultured at the air-liquid interface for 5 days to allow differentiation. **B** Quantification of nuclei in stratum corneum, indicating parakeratosis, n = 10. Bar graph shows mean ± SD; significant differences determined by one-way ANOVA (*p < 0.05; **p < 0.01; ***p < 0.005; ****p < 0.001). **C** Representative images of SE stained with *anti*-KRT10, *anti*-KRT14, and Hoechst nuclear counterstaining. **D** Boxplot showing quantification of epidermal thickness, n = 4. Data are represented as median ± interquartile range; significance was determined by one-way ANOVA. **E** Protein expression of cell cycle inhibition marker P16 and housekeeping marker TUBULIN from the epidermis of the SE. **F** Bar chart showing quantification of P16 vol intensity normalized to TUBULIN with mean ± SD, n = 4, differences determined by one-way ANOVA. **G** Scatter plots showing expression levels of *LMNB1, CDKN1A*, and *HSPA1A* relative to *B2M* from epidermis and dermis of SE, n = 6. Asterisks represent significant differences determined by one-way ANOVA. **H** Confocal images of SE with *anti*-LMNB1 immunostaining and Hoechst nuclear counterstaining. **I** Boxplot showing quantification of LaminB1 mean signal intensity per pixel, measured in a virtual cross section through the epidermal cells, n = 4, significance determined by one-way ANOVA. **J** Confocal images of SE with *anti*-γH2AX immunostaining and Hoechst counterstaining. **K** Bar chart showing quantification of γH2AX positive nuclei by tissue cytometry analysis of the entire tissue section, mean ± SD, n = 3. Significant differences (*p < 0.05; **p < 0.01) determined by Student's t*-*test. Scale bars: A = 50 μm; C = 100 μm; H, J = 20 μm.

To investigate cellular adaptations to a lipid modified matrix microenvironment in the tissue context, we modified a skin equivalent (SE) model system [40] to contain modified collagen in its dermal equivalent compartment. After modification of collagen, we seeded fibroblasts within and keratinocytes on top of the dermal equivalent. Hematoxylin and eosin staining (Fig. 8A) revealed parakeratosis characterized by significantly increased presence of cell nuclei present in the stratum corneum of SE containing with C-OxPAPC (Fig. 8B).

Next, we stained for KRT10 and KRT14 expression and found a disturbed differentiation pattern when the reconstructed SE were generated using modified collagen (Fig. 8C). Epidermal thickness was significantly reduced in SE with HNE modified collagen (Fig. 8D).

To investigate whether senescence associated lipids adducted to collagen would promote senescence of embedded cells, we analyzed a series of established markers [7]. Western blot analysis of epidermal protein expression of P16 (Fig. 8E, full blots in Supplementary Fig. 7) showed significantly higher levels in SE with OxPAPC modified collagen (Fig. 8F).

Gene expression of *CDKN1A* and *HSPA1A* was borderline significant in SE constructed with C-HNE (Fig. 8G). Both modifications led to lower *LMNB1* gene expression levels in the epidermis. Moreover, we performed immunohistochemistry staining of LaminB1 which revealed diminished perinuclear lamina protein expression (Fig. 8H). We confirmed significantly lower expression especially in the epidermis of SE grown on the HNE and OxPAPC modified collagen (Fig. 8I). In addition, we stained against γ H2AX, a DNA damage marker that is commonly associated with senescence (Fig. 8J). Skin equivalents with OxPAPC modified collagen led to significantly higher number of γ H2AX positive nuclei as quantified by tissue cytometry (Fig. 8K, quantification details in Supplementary Fig. 8).

## 4 Discussion

The reactivity of lipid peroxidation products, especially of the prototypic HNE has been extensively studied since lipotoxic and other biological properties of the reactive aldehydes have been first discovered [[56], [57], [58]]. These studies about adduct formation of unsaturated carbonyls with nucleophilic groups on proteins and DNA have spurred the interest in the biological functions and consequences of lipid aldehyde-protein modifications. Recent advances in the mass spectrometric analytical technologies for analyzing lipids and lipid-protein adducts have revealed the extensive diversity and abundance of this type of posttranslational modifications [59].

In this work, we focused on senescent dermal fibroblasts as sources that secrete or expose such reactive aldehydic lipids as part of the secretome of senescent cells, the SASP [9,12]. While the cyto-/chemokine component of the SASP is mostly regarded as a transient phenomenon that ends with the elimination of the senescent cell, the more stable and localized modifications to the ECM brought on by lipid SASP components add potential long-term consequences. These are that the ECM, which is characterized by very slow turnover, provides, after SASP modification, a surface that distorts immune responses, tissue regeneration and differentiation of resident cells and thus may contribute to an aging phenotype.

### 4.1 Chemical aspects

In this study, we described the properties of lipids and its peroxidation products by using Schiff's reagent to determine reactive carbonyl groups. The mixture of oxidized phospholipids (OxPAPC) gave a less intense signal than the aldehydic phospholipid POVPC alone. Due to the lower content of reactive oxidized species, the Oxyblot signal intensity of OxPAPC incubated with collagen was less intense then with the pure and very reactive HNE. We observed different intensities of the DNPH staining with OxPAPC on collagen type I and IV. One possibility would be that when an *α,* β-unsaturated carbonyl reacts predominately as a Schiff base, DNP cannot bind and produce the signal, in contrast to it binding as a Michael adduct.

Although aldehyde-containing fragmented lipids represent a large subfraction of OxPAPC [60], it is still a mixture of oxidation products. This can be attributed to the composition of OxPAPC, in which a typical preparation contains approximately 20% unoxidized PLs, around 50% truncated oxidized products, 25% oxidized cyclic products and 4.5% oxidized full-length products [61]. However, in that study, OxPAPC was obtained from a provider, whereas most laboratories - including ours - have been generating it through autoxidation of PAPC since before it was commercially available. Although the preparations differ slightly, both yield biologically active mixtures of oxidized phospholipids, as demonstrated by extensive literature. A multi-laboratory evaluation of air oxidized PAPC and relative quantification of lipid peroxidation products (LPPs) revealed that the truncation score can help estimating the oxidation status and it is crucial that the preparation method and oxidation time is always consistent [22].

Published studies have shown that during aging - particularly the final third of the lifespan - the levels of oxidatively modified proteins increase dramatically [62]. Identifying which proteins are modified and quantifying the extent of these modifications is of high relevance for biological insight and clinical translation. However, lipids are complex and highly diverse biomolecules with multiple functions, constituting both the native lipidome and the epilipidome - the latter encompassing non-enzymatically modified lipids, most prominently their oxidized species [30]. There are only estimations possible to assess the whole redox epilipidome and therefore it is challenging to determine the relative amount of oxidized lipid modified proteins.

Oxidative modifications play a significant role in cardiovascular diseases, particularly in the context of atherosclerosis research [63], where ECM modifications differ between unstable/soft and stable/hard plaque types [64,65]. In skin research, so far only Larroque et al. showed formation of HNE adducts to elastin by immunofluorescence staining. [33]. While they focused on UVA exposure as a source of lipid aldehydes, we have discovered previously another source of reactive lipids, being derived from senescent cells. While their study focused on immunofluorescence to detect elastin modifications, we were able to extend this work by additionally providing LC-MS/MS analysis of adducts. This revealed distinct high-molecular-weight (HMW) modifications, mainly oxidized phospholipids, as well as low-molecular-weight (LMW) modifications arising from lipid peroxidation–derived scission, traceable to precise amino acid positions in collagen. Overall, arginine residues were most frequently modified, followed by lysine and, to a lesser extent, histidine, whereas cysteine residues were modified exclusively by HMW adducts. We found the SASP lipid PONPC adducting to arginine and cysteine residues of COL1A1. As selected LMW, we detected carboxymethyl (CML, from glyoxal) and HNE to arginine residues.

Adding further complexity, in other studies it was found that HNE-modified proteins are preferentially degraded by proteasomes. However, when crosslinking occurs, protein aggregates can form that inhibit proteasomal activities [66]. Protein crosslinking can happen when the α,β-unsaturated carbonyl forms a Michael adduct and subsequently reacts with a free aldehyde via Schiff base formation [67,68]. Collagen crosslinking has been linked to various diseases, including osteoporosis, diabetes mellitus, chronic kidney disease, inflammatory bowel disease (Saito & Marumo, 2015), and keratoconus - a corneal disorder (Sharif et al., 2018).

### 4.2 Biological consequences

The central question that emerged was whether, and to what extent, lipid-modified collagen affects the physiology and behavior of residing cells. Previous studies have shown that lipid adducts to proteins can disrupt protein function, inhibit enzymatic activity, and enhance immunogenicity (Domingues et al., 2013). Within the context of atherosclerosis research, modification of the lipid matrix in unstable plaques has been associated with increased inflammation, extracellular matrix (ECM) remodeling, and protein degradation [65]. However, the specific functional consequences of dermal ECM modification on resident skin cells remained unclear - until our study provided new insights into these biological effects.

#### 4.2.1 (Bystander) senescence

Recently, Monroe et al. described the induction of cellular senescence by soluble lipid peroxidation products in human lung fibroblasts and murine adipose stem cells [69]. They postulated that the biogenic lipid-induced senescence (BLIS) is caused by free lipid enals covalently modifying DNA and proteins in target cells. In contrast, we investigated how lipids specifically adducted to collagen would affect cellular properties. We observed striking differences in cellular responses to collagen modified by oxidized phospholipids (OxPAPC), which retain the PL backbone, compared to collagen modified by short-chain products such as HNE. Apart from that, also the time of exposure of cells to the modified collagen causes significant differences. Fibroblasts exposed to HNE modified collagen for 24 h exhibited a senescence phenotype, characterized by low levels of *LMNB1, CDK1* and high levels of *CDKN2A, CDKN1A* encoding for P16, P21 respectively. This also explains the lower proliferation outcome assessed by Ki67 staining, another marker to describe senescence according to the MISCE guidelines [7]. After growth for five days this effect on the gene expression level was less prominent, suggesting that the cells can adapt to long exposure of lipid modified collagen or that unaffected cells then outgrow the senescent ones in culture. Senescence being promoted by the SASP of fibroblasts influencing neighboring cells was reported and termed “bystander senescence” [24].

Taken together, the “secondary” bystander effect by SASP modified collagen presented in our model was that monocytes and epidermal cells displayed an early senescent phenotype on both, LMW (HNE) and HMW (OxPAPC) modified collagen. Dermal fibroblasts reacted primarily to LMW modifications with a transient early senescent phenotype.

#### 4.2.2 Oxidative stress

Photooxidation and subsequent oxidation of phospholipids were reported to induce oxidative stress and *HMOX1* expression [47]. We found that this is not only valid for free bioactive lipids but also when they were bound to collagen. While the HNE adducts were detectable by immunofluorescence in the modified matrix, only limited E06 reactivity could be seen outside of the cells. Both modified matrices however were able to elicit reactive intracellular oxygen species production, thereby causing cell associated lipid peroxidation in fibroblasts (not assessed in the other cell types) and expression of oxidative stress markers in all cell types.

It was reported, that especially free HNE triggered *HMOX1* expression and an unfolded protein response (UPR) in neuronal like cells by ROS overproduction and interfering with mitogen-activated protein kinases (MAPK) signaling pathways [50]. In both fibroblasts and macrophages, we observed significantly elevated levels of *HSPA1A* corroborating that the UPR is also caused by HNE adducted to collagen. When the gene levels of *HSPA1A* encoding for the heat shock protein (Hsp70) are elevated, it can help cells dealing with ROS-damaged proteins and support antioxidant enzymes [70].

Another effect of modified collagen we found is a matrix remodeling response with high expression of matrix metalloproteinases and lower levels of collagen synthesis in fibroblasts. Here, an interesting chronological effect was observed as degradation of collagen got higher (*MMP1, MMP3*) the longer fibroblasts were cultured on the modified collagen. Also, from day 3 the cells produce less collagen (*COL1, COL3*).

However, in keratinocytes cultured on lipid modified collagen IV we found significantly lower expression of *MMP9*, a gelatinase to degrade specifically collagen type IV. Interestingly, it was found that downregulation of *MMP9* correlates with inhibition of the MAPK pathway in keratinocytes [71], which could further lead to reduced cell proliferation, altered cell differentiation and changes in inflammatory responses. This would explain the disturbed differentiation pattern we encountered in our skin equivalents with lipid modified collagen.

Apart from that, less dramatic gene expression changes in keratinocytes cultured on modified collagen were detected. We speculate that keratinocytes can escape the oxidative stress naturally by differentiation and are not such a long time affected by the collagen [72].

The question arises what causes these biological responses. Importantly, we found that a possible explanation are free reactive species that trigger the changed gene expression. Our data yield first hints on the mechanistic side behind the observed biological responses. As the antioxidant with radical scavenging activity, N-Acetylcysteine treatment completely blunted the expression of redox stress-, UPR genes and *TNFA* induced by the collagen modification, we conclude that, at least in macrophages, the gene regulation depended on “secondary” intracellular ROS. This would be compatible with a model where first, the contact with the modified matrix caused intracellular ROS elevation and lipid peroxidation which is required for the gene expression and the TLR4 response, as both can be inhibited by NAC. The antioxidant can act as a reduced glutathione (GSH) precursor and can break thiolated proteins, thus releasing reduced proteins with direct antioxidant activity [73].

Furthermore, signaling through collagen receptor interactions could also be affected by modification. The phalloidin staining revealed a significantly changed spreading area in fibroblasts cultured on modified matrices. This indicates functional consequences of altered collagen-integrin receptor interactions [48]. However, specific integrin interaction and signaling has to be investigated on the protein level in future studies.

#### 4.2.3 Inflammation

It was reported previously that free oxidized phospholipids led to the expression of mostly pro-inflammatory genes in endothelial cells [74]. Later a more differentiated view emerged that suggests both pro- and anti-inflammatory properties of free oxidized phospholipids [75]. Especially the ability of OxPAPC to change the phenotypic polarization and differentiation of macrophages [76] is of interest, as it was recently shown in investigation of skin aging, that macrophages were polarized to an inflammatory phenotype by an aged microenvironment of senescent dermal fibroblasts [23]. Therefore, we wanted to find out how senescent derived lipids like OxPAPC, when they are linked to collagen, would influence monocytes. Our finding that also bound lipids drive expression of cyto-/chemokines and affect macrophage properties adds an additional time horizon to the action of (senescent cell derived but also other) reactive oxidized lipids. This may be an additional contribution of senescent cells to aging associated changes in the immune system, adding to the concept of inflammaging, which suggests a chronic low-level inflammation associated with aging [77]. In our cells cultured on the lipid modified collagen we found increased levels of pro-inflammatory cytokines (*IL1, IL6, TNFA)* and a shift towards cellular senescence, indicating an immunosenescence effect.

Moreover, macrophages treated with free OxPAPC revealed interference with Toll-like receptor 2 (TLR2) and TLR4 signaling by inhibiting TNFα production, IκBα degradation, p38 MAPK phosphorylation and NFκB-dependent reporter activation [44,78]. Lipopolysaccharide (LPS) is recognized by TLR4 on immune and non-immune cells. Free OxPAPC was described to have an anti-inflammatory effect in endothelial cells by antagonistic effects on TLR and suppression of RhoA GTPase signaling [79]. In macrophages grown on OxPAPC modified collagen treated with an LPS stimulus, we encountered that the response to LPS regarding induced expression of *TNFA* and with HNE modification also in *CXCL1* and *IL1A* was blunted, indicating an interference with TLR signaling in cells attached to modified matrix.

In more detail, we investigated the response of macrophages cultured on modified matrices to other Toll-like receptors. We found that FSL-1 and Flagellin induced *TNFA* expression was similarly blunted on C-HNE, whereas TNF*α* induced expression of *TNFA* was not inhibited. Interestingly, FSL-1 and Flagellin induced expression of *IL1A* was not inhibited by C-HNE, suggesting a more complex regulation that is not only governed by the C-HNE interaction with the PRR.

Recently, a review explored the mechanisms by which HNE interferes with immune cells [80]. Similar to OxPAPC, the LPO product HNE interacts with several signaling pathways, including NF-κB, Nrf2, MAPK, TLR4 and stimulates interferon genes (STING), thereby affecting immune responses and modulating cytokine production and inflammasome activation. Our findings of collagen modified by HNE support these discoveries and indicate that immunomodulatory effects are possible even after Michael adduction to proteins.

However, *IL8* expression was altered by LPS but not inhibited by growth on C-OxPAPC. It was found previously that drug-like HSP inducers also prevented the induction of IL8 by OxPAPC [81].

### 4.3 Limitations

While our study added principal insights on how senescent cells can affect other skin resident cells via lipid aldehyde driven modification of ECM components, there are clear limits of the scope of this work. We focused on selected collagens and selected lipid aldehydes as partners of these reactions. While the chosen molecules are likely among the most abundant present in the tissue, this is still a limitation for practical and analytical reasons, and we can only assume that also other extracellular matrix proteins and lipid aldehydes can be partners in such a modification reaction. More comprehensive insights will soon be possible with untargeted mass spectrometric adductomic analysis, as will be the quantitative aspects of our findings *in vivo*.

In this study we performed first experiments to find underlying functional causes for the responses of the cells. In macrophages, we identified “secondary” ROS generation as trigger for changes induced by the modified matrix, but the mechanistic setting and the cell surface molecules and signaling cascades behind the findings should be explored in more detail in future studies in all involved cell types and in tissue.

### 4.4 Conclusions

Taken together, our data suggests that oxidized lipid modifications of collagen could be a contribution of the SASP to specific aspects of skin aging. Our dermal equivalent model with lipids adducted to collagen could help investigate senescence and age-associated diseases.

As adducted high- and low-molecular weight lipid aldehydes interfere with pattern recognition receptor signaling, our finding could suggest that modified matrix reduces the immune surveillance. Thereby the reduced ability of phagocytes to recognize oxidation/senescence associated patterns may contribute to immune evasion of entities which carry oxidized epitopes, including senescent cells themselves. Whether the lipid modified collagen differs in integrin signaling and whether physical properties of collagen like elasticity and stability are changed would merit further investigation to gain a more holistic view on cell to ECM interaction. Therefore, it should be further investigated how modified collagen interferes with wound healing and which role it plays in various skin diseases like fibrosis or cancer.

Since we found that treatment with the antioxidant NAC revoked the biological consequences of the lipid modified collagen, the use of this or similar compounds may have unexpected translational potential in targeting secondary negative effects of senescent cells and their SASP that are mediated by modified matrix.

In conclusion, our study advances the understanding of the biological effects of oxidized lipids adducted to ECM proteins and their implications for the functions of resident cells.

## Funding sources

FG gratefully acknowledges the financial support of the Federal Ministry of Economy, Energy and Tourism of Austria and the National Foundation for Research, Technology, and Development of Austria and of Chanel P. B. to the Christian Doppler Laboratory for Skin Multimodal Imaging of Aging and Senescence. Part of the work was supported by supported by the FWF I-5627 grant to F.G.

Work in the Fedorova lab is supported by ‘‘Sonderzuweisung zur Unterstützung profilbestimmender Struktureinheiten’’ by the SMWK to 10.13039/501100002957TUD, TG70 by Sächsische Aufbaubank and 10.13039/501100006114SMWK, the measure is co-financed with tax funds on the basis of the budget passed by the Saxon state parliament (to M.F.), 10.13039/501100001659Deutsche Forschungsgemeinschaft (FE 1236/5-1, FE 1236/8-1 to M.F.), and Bundesministerium für Bildung und Forschung (031L0315A, DEEP_HCC and 01EJ2205A, FERROPath to M.F.).

This article/publication is partially based on work from 10.13039/501100000921COST Action EpiLipidNET, CA19105, supported by 10.13039/501100000921COST (10.13039/501100000921European Cooperation in Science and Technology).

## CRediT authorship contribution statement

**Sarah Jelleschitz:** Formal analysis, Investigation, Methodology, Writing – original draft. **Christopher Kremslehner:** Formal analysis, Investigation, Methodology, Visualization. **Ionela-Mariana Nagelreiter:** Investigation, Methodology, Resources. **Michael Mildner:** Methodology. **Melanie Salek:** Formal analysis, Investigation, Methodology. **Christina Bauer:** Formal analysis, Methodology, Visualization. **Alexandra Stiegler:** Methodology, Resources. **Adrian Sandgren Fors:** Investigation, Methodology. **Michaela Schirato:** Data curation, Formal analysis, Investigation, Methodology, Supervision. **Christian Freystätter:** Resources. **Agnès Tessier:** Methodology, Resources, Writing – review & editing. **Francesca Marcato:** Resources, Writing – review & editing. **Gaëlle Gendronneau:** Investigation, Supervision. **Nada André:** Writing – review & editing. **Youcef Ben Khalifa:** Resources, Supervision. **Zhixu Ni:** Data curation, Formal analysis, Investigation, Methodology. **Maria Fedorova:** Data curation, Investigation, Methodology, Resources, Validation, Writing – original draft. **Olga Oskolkova:** Conceptualization, Methodology, Writing – review & editing. **Marie-Sophie Narzt:** Formal analysis, Investigation, Validation. **Florian Gruber:** Conceptualization, Funding acquisition, Supervision, Writing – review & editing.

## Declaration of competing interest

The authors declare the following financial interests/personal relationships which may be considered as potential competing interests: Agnès Tessier, Francesca Marcato, Gaëlle Gendronneau, Nada Andre and Youcef Ben Khalifa are employees of Chanel Parfums Beauté, 8 rue du Cheval Blanc 93500 Pantin, France.

## Data Availability

Data will be made available on request.

## Abbreviations

B2M: β-2-microglobulin
CDK1: Cyclin-dependent kinase 1
CDKN1A, P21: Cyclin-dependent kinase inhibitor 1A
CDKN2A, P16: Cyclin-dependent kinase inhibitor 2A
CML: Carboxymethyl lysine
CXCL1: Chemokine (C-X-C motif) ligand 1
DNPH: 2,4-Dinitrophenylhydrazine
DPPC: Dipalmitoyl-*sn*-glycero-3-phosphocholine
ECM: Extracellular matrix
FB: Fibroblasts
FSL-1: Fibroblast-stimulating Lipopeptide-1
GCLM: Glutamate-cysteine ligase
HMOX1: Heme oxygenase 1
HMW: High molecular weight
HNE: 4-Hydroxnonenal
HSPA1A: Heat shock protein family A (Hsp70) member 1A
IL: Interleukin
KC: Keratinocytes
LC-MS/MS: Liquid chromatography-tandem mass spectrometry
LMW: Low molecular weight
LPO: Lipid peroxidation
LPS: Lipopolysaccharide
MA: Michael addition
MMP: Matrix metalloproteinase
NAC: N-Acetylcysteine
OxPAPC: Oxidized1-Palmitoyl-2-arachidonoyl-*sn*-glycero-3-phosphocholine
PAPC: 1-Palmitoyl-2-arachidonoyl-*sn*-glycero-3-phosphocholine
PL: Phospholipid
PMA: Phorbol-12-myristate-13-acetate
Poly(I:C): Polyinosinic-polycytidylic acid (Poly(I:C)
PONPC: 1-Palmitoyl-2-(9′-oxo-nonanoyl)-*sn*-glycero-3-phosphocholine
POVPC: 1-Palmitoyl-2-(5′-oxo-valeroyl)-*sn*-glycero-3-phosphocholine
ROS: Reactive Oxygen Species
SASP: Senescence-associated secretory phenotype
SB: Schiff base
SIPS: Stress induced premature senescence
TLR: Toll-like receptor
TNFα: Tumor necrosis factor α

## Appendix A Supplementary data

Multimedia component 2 Multimedia component 2

Multimedia component 3 Supplementary Figure 1. A Timeline of experimental procedure for culturing of fibroblasts (FB), macrophages (MΦ) and keratinocytes (KC) on collagen. **B** Immunostaining of cell free matrices of *in vitro* modified collagen with HNE and OxPAPC detected with *anti*-HNE Michael adducts antibody and *anti*-E06 antibody, respectively. **C** Immunostaining of human skin (f30y) irradiated for three weeks with a total dose of 600 J/cm^2^ UVA. Merged and split channel images show colocalization of collagen type I with *anti*-HNE Michael adducts antibody. **D** Western Blot of protein extracted separately from the epidermis and dermis of the irradiated skin. Bar chart shows quantification of MMP1 volume intensity normalized to the TUBULIN signal. Supplementary Figure 2. A Representative images of phalloidin staining analysis. Strata-Quest tissue cytometry software was used to detect nuclei stained with Hoechst and generate a corresponding cell mask around the phalloidin staining of actin filaments. The detected cell perimeter is visualized by white lines. **B** Fibroblasts (f33y) were cultured for 24 h on modified collagen in biological quadruplicates. Gene expression was analyzed by qPCR and quantified relative to *B2M*. Bar charts show the relative expression level; asterisks represent significant differences (**p* < 0.05; ***p* < 0.01; ****p* < 0.005; *****p* < 0.001) of the mean determined by one-way ANOVA. Supplementary Figure 3 ztarttnqtech. Fibroblasts cultured for up to 5 days on modified collagen type I. **A** Images show fibroblasts on different matrices after growth of 5 days, scale bars = 200 μm. **A, B** Scattered plots show the expression levels relative to *B2M*. Quadruplicates of fibroblasts from four donors (m27y, f34y, m32y, f41y) were used, n = 16. Asterisks represent significant differences (**p* < 0.05; ***p* < 0.01; ****p* < 0.005) of the mean determined by one-way ANOVA. Supplementary Figure 4. THP-1 cells were treated with 10 nM PMA and grown for 24 h on collagen. 500 ng/ml LPS were added to the macrophages for 2 h to determine the LPS effect. Plain colored bar charts show relative expression level and dotted bars represent results of the LPS stimulus quantified to *B2M,* n = 4. Asterisks represent significant differences (**p* < 0.05; ***p* < 0.01; ****p* < 0.005) determined by one-way ANOVA. Supplementary Figure 5. A Bar charts with solid-colored bars represent the effect of the modified matrix on macrophages. THP-1 cells were treated with 10 nM PMA and cultured on modified collagen for 24 h. Expression levels were analyzed relative to *EEF1A1*, bar charts show mean ± SD, n = 4. **B** Bar charts with dotted bars represent the gene expression relative to *EEF1A1* after the LPS stimulation (TLR4 agonist). Cells were treated with 500 ng/ml LPS for 2 h, n = 4. **C** Bar charts show relative expression levels of macrophages, quantified to *EEF1A1*. Cells were treated with 5 mM N-Acetylcysteine (NAC) immediately after seeding and cultured for 24 h, followed by stimulation with 500 ng/ml LPS for 2 h, mean ± SD, n = 4. Asterisks indicate statistical significance (**p* < 0.05; ***p* < 0.01; ****p* < 0.005) determined by one-way ANOVA. Supplementary Figure 6 ztarttnqtech. Keratinocytes cultured for up to 72 h on modified human collagen type IV. **A** Images show keratinocytes on different matrices after growth for 24 h, scale bars = 200 μm. **A, B** Scattered plots show the expression levels relative to *B2M*. Quadruplicates of keratinocytes from two donors (f38y and m27y) were used, n = 8. Asterisks represent significant differences (**p* < 0.05; ***p* < 0.01; ****p* < 0.005) of the mean determined by one-way ANOVA. Supplementary Figure 7. Full Western Blot pictures of P16 and TUBULIN protein extracted from the epidermis of the skin equivalents using two keratinocyte donors (f30y, f35y), n = 4. Supplementary Figure 8 ztarttnqtech. Whole tissue sections were subjected to staining of γH2AX, recorded using TissueFAXS, and analyzed with StrataQuest software. White arrows on the images highlight automatically identified positive cells. The graph depicts gating of γH2AX positive cells per full skin equivalent, shown as a percentage. Representative images of cell nuclei illustrate the applied detection thresholds distinguishing positive and negative nuclei. Multimedia component 3

## References

[1] Eckhart L., Zeeuwen P.L.J.M. (2018). The skin barrier: epidermis vs environment. Exp. Dermatol..

[2] Fisher G.J., Wang B., Cui Y., Shi M., Zhao Y., Quan T., Voorhees J.J. (2023). Skin aging from the perspective of dermal fibroblasts: the interplay between the adaptation to the extracellular matrix microenvironment and cell autonomous processes. J. Cell Commun. Signal..

[3] Yin D. (1996). Biochemical basis of lipofuscin, ceroid, and age pigment-like fluorophores. Free Radic. Biol. Med..

[4] Brunk U.T., Terman A. (2002). Lipofuscin: mechanisms of age-related accumulation and influence on cell function12 1Guest editor: Rajindar S. sohal 2This article is part of a series of reviews on “Oxidative Stress and Aging.” the full list of papers may be found on the homepage of the. Free Radic. Biol. Med..

[5] Ho C.Y., Dreesen O. (2021). Faces of cellular senescence in skin aging. Mech. Ageing Dev..

[6] Gruber F., Kremslehner C., Eckhart L., Tschachler E. (2020). Cell aging and cellular senescence in skin aging — recent advances in fibroblast and keratinocyte biology. Exp. Gerontol..

[7] Ogrodnik M., Carlos Acosta J., Adams P.D., D'Adda Di Fagagna F., Baker D.J., Bishop C.L., Chandra T., Collado M., Gil J., Gorgoulis V., Gruber F., Hara E., Jansen-Dürr P., Jurk D., Khosla S., Kirkland J.L., Krizhanovsky V., Minamino T., Niedernhofer L.J., Demaria M. (2024). Guidelines for minimal information on cellular senescence experimentation in vivo. Cell.

[8] Özcan S., Alessio N., Acar M.B., Mert E., Omerli F., Peluso G., Galderisi U. (2016). Unbiased analysis of senescence associated secretory phenotype (SASP) to identify common components following different genotoxic stresses. Aging.

[9] Ni C., Narzt M.-S., Nagelreiter I.-M., Zhang C.F., Larue L., Rossiter H., Grillari J., Tschachler E., Gruber F. (2016). Autophagy deficient melanocytes display a senescence associated secretory phenotype that includes oxidized lipid mediators. Int. J. Biochem. Cell Biol..

[10] Greenberg M.E., Li X.-M., Gugiu B.G., Gu X., Qin J., Salomon R.G., Hazen S.L. (2008). The lipid whisker model of the structure of oxidized cell membranes. J. Biol. Chem..

[11] Binder C.J., Papac-Milicevic N., Witztum J.L. (2016). Innate sensing of oxidation-specific epitopes in health and disease. Nat. Rev. Immunol..

[12] Narzt M.S., Pils V., Kremslehner C., Nagelreiter I.M., Schosserer M., Bessonova E., Bayer A., Reifschneider R., Terlecki-Zaniewicz L., Waidhofer-Sollner P., Mildner M., Tschachler E., Cavinato M., Wedel S., Jansen-Durr P., Nanic L., Rubelj I., El-Ghalbzouri A., Zoratto S., Lammermann I. (2021). Epilipidomics of senescent dermal fibroblasts identify lysophosphatidylcholines as pleiotropic senescence-associated secretory phenotype (SASP) factors. J. Invest. Dermatol..

[13] Flor A.C., Wolfgeher D., Wu D., Kron S.J. (2017). A signature of enhanced lipid metabolism, lipid peroxidation and aldehyde stress in therapy-induced senescence. Cell Death Discov..

[14] Fritz K.S., Petersen D.R. (2013). An overview of the chemistry and biology of reactive aldehydes. Free Radic. Biol. Med..

[15] Singh M., Kapoor A., Bhatnagar A. (2015). Oxidative and reductive metabolism of lipid-peroxidation derived carbonyls. Chem. Biol. Interact..

[16] Cordes E.H., Jencks W.P. (1962). On the mechanism of schiff base formation and hydrolysis. J. Am. Chem. Soc..

[17] Jackson P.A., Widen J.C., Harki D.A., Brummond K.M. (2017). Covalent modifiers: a chemical perspective on the reactivity of α,β-Unsaturated carbonyls with thiols via hetero-michael addition reactions. J. Med. Chem..

[18] Viedma-Poyatos Á., González-Jiménez P., Langlois O., Company-Marín I., Spickett C.M., Pérez-Sala D. (2021). Protein lipoxidation: basic concepts and emerging roles. Antioxidants.

[19] Kim C.H., Zou Y., Kim D.H., Kim N.D., Yu B.P., Chung H.Y. (2006). Proteomic analysis of nitrated and 4-Hydroxy-2-Nonenal-Modified serum proteins during aging. J. Gerontol., Ser. A Biol. Sci. Med. Sci..

[20] Vazdar K., Škulj S., Bakarić D., Margetić D., Vazdar M. (2021). Chemistry and reactivity of 4-hydroxy-2-nonenal (HNE) in model biological systems. Mini Rev. Med. Chem..

[21] Berliner J. (2001). Evidence for a role of phospholipid oxidation products in atherogenesis. Trends Cardiovasc. Med..

[22] Ni Z., Sousa B.C., Colombo S., Afonso C.B., Melo T., Pitt A.R., Spickett C.M., Domingues P., Domingues M.R., Fedorova M., Criscuolo A. (2019). Evaluation of air oxidized PAPC: a multi laboratory study by LC-MS/MS. Free Radic. Biol. Med..

[23] Gather L., Nath N., Falckenhayn C., Oterino-Sogo S., Bosch T., Wenck H., Winnefeld M., Grönniger E., Simm S., Siracusa A. (2022). Macrophages are polarized toward an inflammatory phenotype by their aged microenvironment in the human skin. J. Invest. Dermatol..

[24] Nelson G., Wordsworth J., Wang C., Jurk D., Lawless C., Martin‐Ruiz C., Von Zglinicki T. (2012). A senescent cell bystander effect: senescence‐induced senescence. Aging Cell.

[25] Robins J.H., Abrams G.D., Pincock J.A. (1980). The structure of schiff reagent aldehyde adducts and the mechanism of the schiff reaction as determined by nuclear magnetic resonance spectroscopy. Can. J. Chem..

[26] Tsai L., Szweda P.A., Vinogradova O., Szweda L.I. (1998). Structural characterization and immunochemical detection of a fluorophore derived from 4-hydroxy-2-nonenal and lysine. Proc. Natl. Acad. Sci..

[27] Bird D.A., Gillotte K.L., Hörkkö S., Friedman P., Dennis E.A., Witztum J.L., Steinberg D. (1999). Receptors for oxidized low-density lipoprotein on elicited mouse peritoneal macrophages can recognize both the modified lipid moieties and the modified protein moieties: implications with respect to macrophage recognition of apoptotic cells. Proc. Natl. Acad. Sci..

[28] Pégorier S., Stengel D., Durand H., Croset M., Ninio E. (2006). Oxidized phospholipid: POVPC binds to platelet-activating-factor receptor on human macrophages. Atherosclerosis.

[29] König J., Ott C., Hugo M., Jung T., Bulteau A.-L., Grune T., Höhn A. (2017). Mitochondrial contribution to lipofuscin formation. Redox Biol..

[30] Wölk M., Prabutzki P., Fedorova M. (2023). Analytical toolbox to unlock the diversity of oxidized lipids. Acc. Chem. Res..

[31] Iram N., Mildner M., Prior M., Petzelbauer P., Fiala C., Hacker S., Schöppl A., Tschachler E., Elbe-Bürger A. (2012). Age-related changes in expression and function of toll-like receptors in human skin. Development.

[32] Bormann D., Copic D., Klas K., Direder M., Riedl C.J., Testa G., Kühtreiber H., Poreba E., Hametner S., Golabi B., Salek M., Haider C., Endmayr V., Shaw L.E., Höftberger R., Ankersmit H.J., Mildner M. (2023). Exploring the heterogeneous transcriptional response of the CNS to systemic LPS and Poly(I:C). Neurobiol. Dis..

[33] Larroque-Cardoso P., Camaré C., Nadal-Wollbold F., Grazide M.-H., Pucelle M., Garoby-Salom S., Bogdanowicz P., Josse G., Schmitt A.-M., Uchida K., Zarkovic K., Salvayre R., Nègre-Salvayre A. (2015). Elastin modification by 4-Hydroxynonenal in hairless mice exposed to UV-A. Role in photoaging and actinic elastosis. J. Invest. Dermatol..

[34] Vile G.F., Tyrrell R.M. (1995). Uva radiation-induced oxidative damage to lipids and proteins in vitro and in human skin fibroblasts is dependent on iron and singlet oxygen. Free Radic. Biol. Med..

[35] Uchida K., Toyokuni S., Nishikawa K., Kawakishi S., Oda H., Hiai H., Stadtman E.R. (1994). Michael addition-type 4-Hydroxy-2-nonenal adducts in modified low-density lipoproteins: markers for atherosclerosis. Biochemistry.

[36] Hörkkö S., Bird D.A., Miller E., Itabe H., Leitinger N., Subbanagounder G., Berliner J.A., Friedman P., Dennis E.A., Curtiss L.K., Palinski W., Witztum J.L. (1999). Monoclonal autoantibodies specific for oxidized phospholipids or oxidized phospholipid–protein adducts inhibit macrophage uptake of oxidized low-density lipoproteins. J. Clin. Investig..

[37] Sochorová M., Kremslehner C., Nagelreiter I.M., Ferrara F., Lisicin M.M., Narzt M.S., Bauer C., Stiegler A., Golabi B., Vávrová K., Gruber F. (2023). Deletion of <scp>NRF2</scp> disturbs composition, morphology, and differentiation of the murine tail epidermis in chronological aging. Biofactors.

[38] Gruber F., Mayer H., Lengauer B., Mlitz V., Sanders J.M., Kadl A., Bilban M., Martin R., Wagner O., Kensler T.W., Yamamoto M., Leitinger N., Tschachler E. (2010). NF‐E2‐related factor 2 regulates the stress response to UVA‐1‐oxidized phospholipids in skin cells. FASEB J..

[39] Pfaffl M.W. (2001). A new mathematical model for relative quantification in real-time RT-PCR. Nucleic Acids Res..

[40] Mildner M., Jin J., Eckhart L., Kezic S., Gruber F., Barresi C., Stremnitzer C., Buchberger M., Mlitz V., Ballaun C., Sterniczky B., Födinger D., Tschachler E. (2010). Knockdown of filaggrin impairs diffusion barrier function and increases UV sensitivity in a human skin model. J. Invest. Dermatol..

[41] Gruber F., Marchetti-Deschmann M., Kremslehner C., Schosserer M. (2021). The skin epilipidome in stress, aging, and inflammation. Front. Endocrinol..

[42] Hamsanathan S., Gurkar A.U. (2022). Lipids as regulators of cellular senescence. Front. Physiol..

[43] Leitinger N., Tyner T.R., Oslund L., Rizza C., Subbanagounder G., Lee H., Shih P.T., Mackman N., Tigyi G., Territo M.C., Berliner J.A., Vora D.K. (1999). Structurally similar oxidized phospholipids differentially regulate endothelial binding of monocytes and neutrophils. Proc. Natl. Acad. Sci..

[44] Bochkov V.N., Kadl A., Huber J., Gruber F., Binder B.R., Leitinger N. (2002). Protective role of phospholipid oxidation products in endotoxin-induced tissue damage. Nature.

[45] Bochkov V.N., Oskolkova O.V., Birukov K.G., Levonen A.-L., Binder C.J., Stöckl J. (2010). Generation and biological activities of oxidized phospholipids. Antioxidants Redox Signal..

[46] Witztum J., Binder C., Chou M.-Y., Fogelstrand L., Hartvigsen K., Shaw P., Boullier A. (2008). Natural antibodies in murine atherosclerosis. Curr. Drug Targets.

[47] Gruber F., Oskolkova O., Leitner A., Mildner M., Mlitz V., Lengauer B., Kadl A., Mrass P., Krönke G., Binder B.R., Bochkov V.N., Leitinger N., Tschachler E. (2007). Photooxidation generates biologically active phospholipids that induce heme Oxygenase-1 in skin cells. J. Biol. Chem..

[48] Segal G., Lee W., Arora P.D., McKee M., Downey G., McCulloch C.A.G. (2001). Involvement of actin filaments and integrins in the binding step in collagen phagocytosis by human fibroblasts. J. Cell Sci..

[49] Langholz O., Röckel D., Mauch C., Kozlowska E., Bank I., Krieg T., Eckes B. (1995). Collagen and collagenase gene expression in three-dimensional collagen lattices are differentially regulated by alpha 1 beta 1 and alpha 2 beta 1 integrins. J. Cell Biol..

[50] Lin M.-H., Yen J.-H., Weng C.-Y., Wang L., Ha C.-L., Wu M.-J. (2014). Lipid peroxidation end product 4-hydroxy-trans-2-nonenal triggers unfolded protein response and heme oxygenase-1 expression in PC12 cells: roles of ROS and MAPK pathways. Toxicology.

[51] Afonyushkin T., Oskolkova O.V., Binder B.R., Bochkov V.N. (2011). Involvement of CK2 in activation of electrophilic genes in endothelial cells by oxidized phospholipids. JLR (J. Lipid Res.).

[52] Lee J.Y., Hwang D.H. (2006). The modulation of inflammatory gene expression by lipids: mediation through toll-like receptors. Mol. Cells.

[53] Von Schlieffen E., Oskolkova O.V., Schabbauer G., Gruber F., BlüMl S., Genest M., Kadl A., Marsik C., Knapp S., Chow J., Leitinger N., Binder B.R., Bochkov V.N. (2009). Multi-hit inhibition of circulating and cell-associated components of the toll-like receptor 4 pathway by oxidized phospholipids. Arterioscler. Thromb. Vasc. Biol..

[54] Gargiulo S., Gamba P., Testa G., Rossin D., Biasi F., Poli G., Leonarduzzi G. (2015). Relation between TLR4/NF‐κB signaling pathway activation by 27‐hydroxycholesterol and 4‐hydroxynonenal, and atherosclerotic plaque instability. Aging Cell.

[55] Liu Y., Ho C., Wen D., Sun J., Huang L., Gao Y., Li Q., Zhang Y. (2022). Targeting the stem cell niche: role of collagen XVII in skin aging and wound repair. Theranostics.

[56] Esterbauer H., Schaur R.J., Zollner H. (1991). Chemistry and biochemistry of 4-hydroxynonenal, malonaldehyde and related aldehydes. Free Radic. Biol. Med..

[57] Petersen D.R., Doorn J.A. (2004). Reactions of 4-hydroxynonenal with proteins and cellular targets. Free Radic. Biol. Med..

[58] Sousa B.C., Pitt A.R., Spickett C.M. (2017). Chemistry and analysis of HNE and other prominent carbonyl-containing lipid oxidation compounds. Free Radic. Biol. Med..

[59] Spickett C.M., Reis A., Pitt A.R. (2013). Use of narrow mass-window, high-resolution extracted product ion chromatograms for the sensitive and selective identification of protein modifications. Anal. Chem..

[60] Birukova A.A., Starosta V., Tian X., Higginbotham K., Koroniak L., Berliner J.A., Birukov K.G. (2013). Fragmented oxidation products define barrier disruptive endothelial cell response to OxPAPC. Transl. Res..

[61] Camunas-Alberca S.M., Taha A.Y., Gradillas A., Barbas C. (2025). Comprehensive analysis of oxidized arachidonoyl-containing glycerophosphocholines using ion mobility spectrometry-mass spectrometry. Talanta.

[62] Levine R.L., Stadtman E.R. (2001). Oxidative modification of proteins during aging. Exp. Gerontol..

[63] Stocker R., Keaney J.F. (2004). Role of oxidative modifications in atherosclerosis. Physiol. Rev..

[64] Yang-Jensen K.C., Jørgensen S.M., Chuang C.Y., Davies M.J. (2024). Modification of extracellular matrix proteins by oxidants and electrophiles. Biochem. Soc. Trans..

[65] Lorentzen L.G., Yeung K., Eldrup N., Eiberg J.P., Sillesen H.H., Davies M.J. (2024). Proteomic analysis of the extracellular matrix of human atherosclerotic plaques shows marked changes between plaque types. Matrix Biol..

[66] Grune T., Davies K.J.A. (2003). The proteasomal system and HNE-Modified proteins. Mol. Aspect. Med..

[67] Friguet B., Stadtman E.R., Szweda L.I. (1994). Modification of glucose-6-phosphate dehydrogenase by 4-hydroxy-2-nonenal. Formation of cross-linked protein that inhibits the multicatalytic protease. J. Biol. Chem..

[68] Uchida K., Stadtman E.R. (1993). Covalent attachment of 4-hydroxynonenal to glyceraldehyde-3-phosphate dehydrogenase. A possible involvement of intra- and intermolecular cross-linking reaction. J. Biol. Chem..

[69] Monroe T.B., Hertzel A.V., Dickey D.M., Hagen T., Santibanez S.V., Berdaweel I.A., Halley C., Puchalska P., Anderson E.J., Camell C.D., Robbins P.D., Bernlohr D.A. (2025). Lipid peroxidation products induce carbonyl stress, mitochondrial dysfunction, and cellular senescence in human and murine cells. Aging Cell.

[70] Zhang H., Gong W., Wu S., Perrett S. (2022). Hsp70 in redox homeostasis. Cells.

[71] Holvoet S., Vincent C., Schmitt D., Serres M. (2003). The inhibition of MAPK pathway is correlated with down-regulation of MMP-9 secretion induced by TNF-α in human keratinocytes. Exp. Cell Res..

[72] Vessey D.A., Lee K.-H., Boyer T.D. (1995). Differentiation-induced enhancement of the ability of cultured human keratinocytes to suppress oxidative stress. J. Invest. Dermatol..

[73] Aldini G., Altomare A., Baron G., Vistoli G., Carini M., Borsani L., Sergio F. (2018). N-Acetylcysteine as an antioxidant and disulphide breaking agent: the reasons why. Free Radic. Res..

[74] Kadl A., Huber J., Gruber F., Bochkov V.N., Binder B.R., Leitinger N. (2002). Analysis of inflammatory gene induction by oxidized phospholipids in vivo by quantitative real-time RT-PCR in comparison with effects of LPS. Vasc. Pharmacol..

[75] Bochkov V.N., Leitinger N. (2003). Anti-inflammatory properties of lipid oxidation products. J. Mol. Med..

[76] Serbulea V., Deweese D., Leitinger N. (2017). The effect of oxidized phospholipids on phenotypic polarization and function of macrophages. Free Radic. Biol. Med..

[77] Pilkington S.M., Bulfone-Paus S., Griffiths C.E.M., Watson R.E.B. (2021). Inflammaging and the skin. J. Invest. Dermatol..

[78] Erridge C., Kennedy S., Spickett C.M., Webb D.J. (2008). Oxidized phospholipid inhibition of toll-like receptor (TLR) signaling is restricted to TLR2 and TLR4. J. Biol. Chem..

[79] Ke Y., Zebda N., Oskolkova O., Afonyushkin T., Berdyshev E., Tian Y., Meng F., Sarich N., Bochkov V.N., Wang J.M., Birukova A.A., Birukov K.G. (2017). Anti-inflammatory effects of OxPAPC involve endothelial cell–mediated generation of LXA4. Circ. Res..

[80] Ioannidis M., Tjepkema J., Uitbeijerse M.R.P., Van Den Bogaart G. (2025). Immunomodulatory effects of 4-hydroxynonenal. Redox Biol..

[81] Hellauer K., Oskolkova O.V., Gesslbauer B., Bochkov V. (2023). Pharmacological heat-shock protein inducers and chemical chaperones inhibit upregulation of interleukin-8 by oxidized phospholipids. Inflammopharmacology.

