# Supplementary material for: Modification of the dermal matrix by senescence associated lipids and its functional consequence
Source: Redox Biol. 2026 Feb 10;91:104069. doi: 10.1016/j.redox.2026.104069 (PMC12925199; doi:10.1016/j.redox.2026.104069)
Supplement: Multimedia component 3 — Supplementary Figure 1. A Timeline of experimental procedure for culturing of fibroblasts (FB), macrophages (MΦ) and keratinocytes (KC) on collagen. B Immunostaining of cell free matrices of in vitro modified collagen with HNE and OxPAPC detected with anti-HNE Michael adducts antibody and anti-E06 antibody, respectively. C Immunostaining of human skin (f30y) irradiated for three weeks with a total dose of 600 J/cm2 UVA. Merged and split channel images show colocalization of collagen type I with anti-HNE Michael adducts antibody. D Western Blot of protein extracted separately from the epidermis and dermis of the irradiated skin. Bar chart shows quantification of MMP1 volume intensity normalized to the TUBULIN signal. Supplementary Figure 2. A Representative images of phalloidin staining analysis. Strata-Quest tissue cytometry software was used to detect nuclei stained with Hoechst and generate a corresponding cell mask around the phalloidin staining of actin filaments. The detected cell perimeter is visualized by white lines. B Fibroblasts (f33y) were cultured for 24 h on modified collagen in biological quadruplicates. Gene expression was analyzed by qPCR and quantified relative to B2M. Bar charts show the relative expression level; asterisks represent significant differences (*p < 0.05; **p < 0.01; ***p < 0.005; ****p < 0.001) of the mean determined by one-way ANOVA. Supplementary Figure 3 ztarttnqtech. Fibroblasts cultured for up to 5 days on modified collagen type I. A Images show fibroblasts on different matrices after growth of 5 days, scale bars = 200 μm. A, B Scattered plots show the expression levels relative to B2M. Quadruplicates of fibroblasts from four donors (m27y, f34y, m32y, f41y) were used, n = 16. Asterisks represent significant differences (*p < 0.05; **p < 0.01; ***p < 0.005) of the mean determined by one-way ANOVA. Supplementary Figure 4. THP-1 cells were treated with 10 nM PMA and grown for 24 h on collagen. 500 ng/ml LPS were added to the macrop [file mmc3.pptx]

## Slide 1
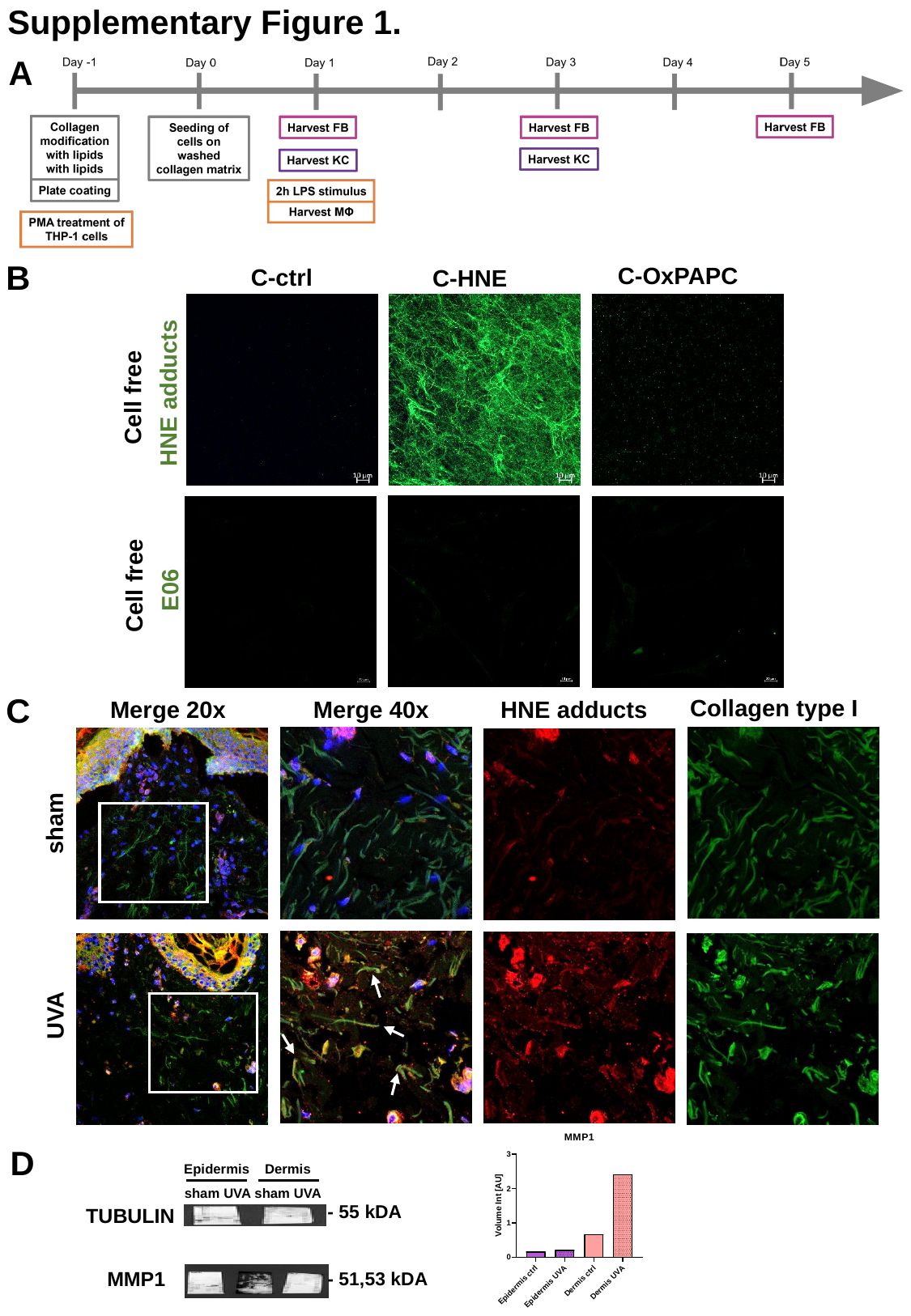

Supplementary Figure 1.
A
B
C-OxPAPC
C-ctrl
C-HNE
HNE adducts
Cell free
E06
Cell free
C
Collagen type I
HNE adducts
Merge 20x
Merge 40x
sham
UVA
D
Epidermis Dermis
sham UVA sham UVA
- 55 kDA
TUBULIN
MMP1
- 51,53 kDA

## Slide 2
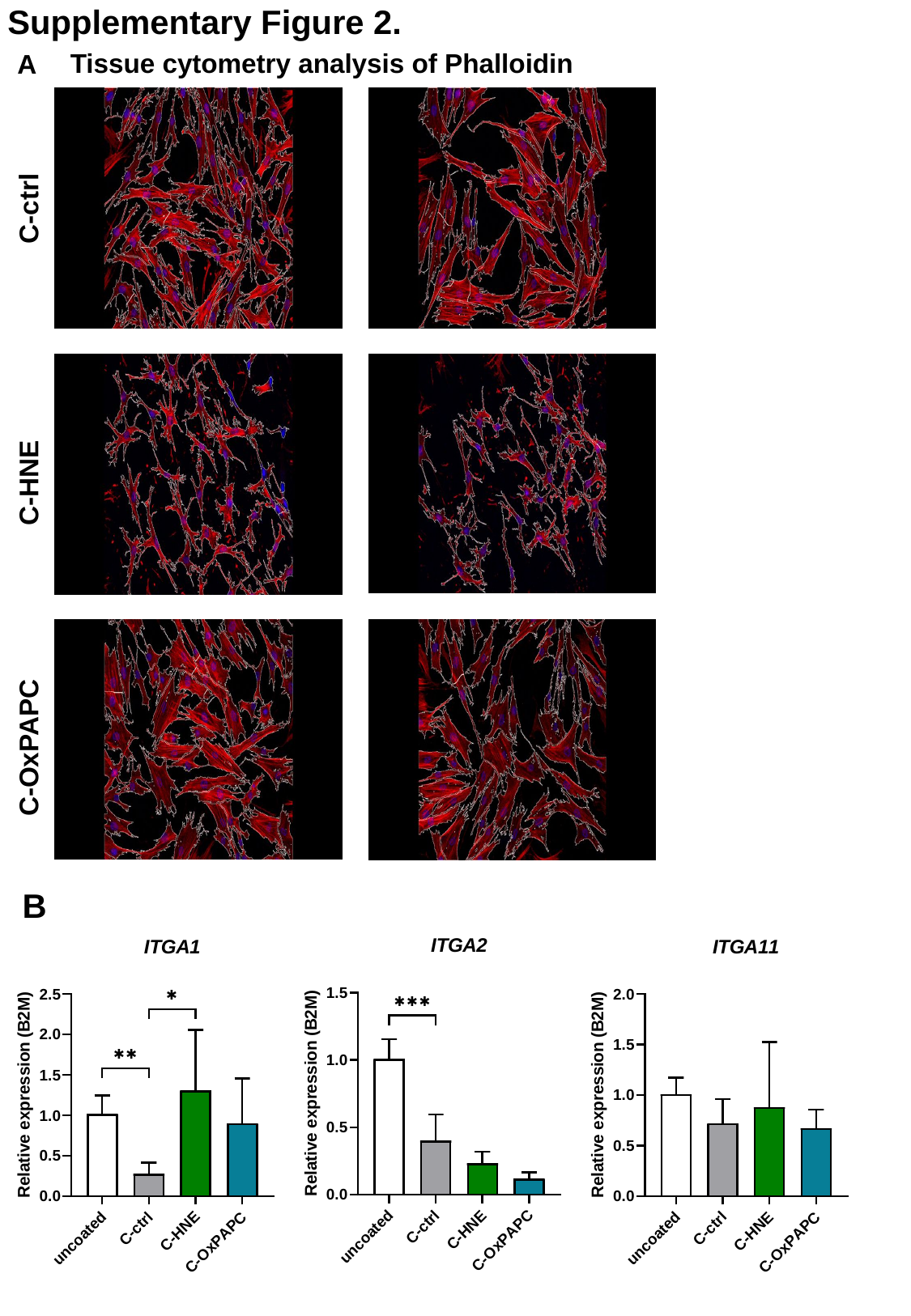

Supplementary Figure 2.
Tissue cytometry analysis of Phalloidin
A
C-ctrl
C-HNE
C-OxPAPC
B

## Slide 3
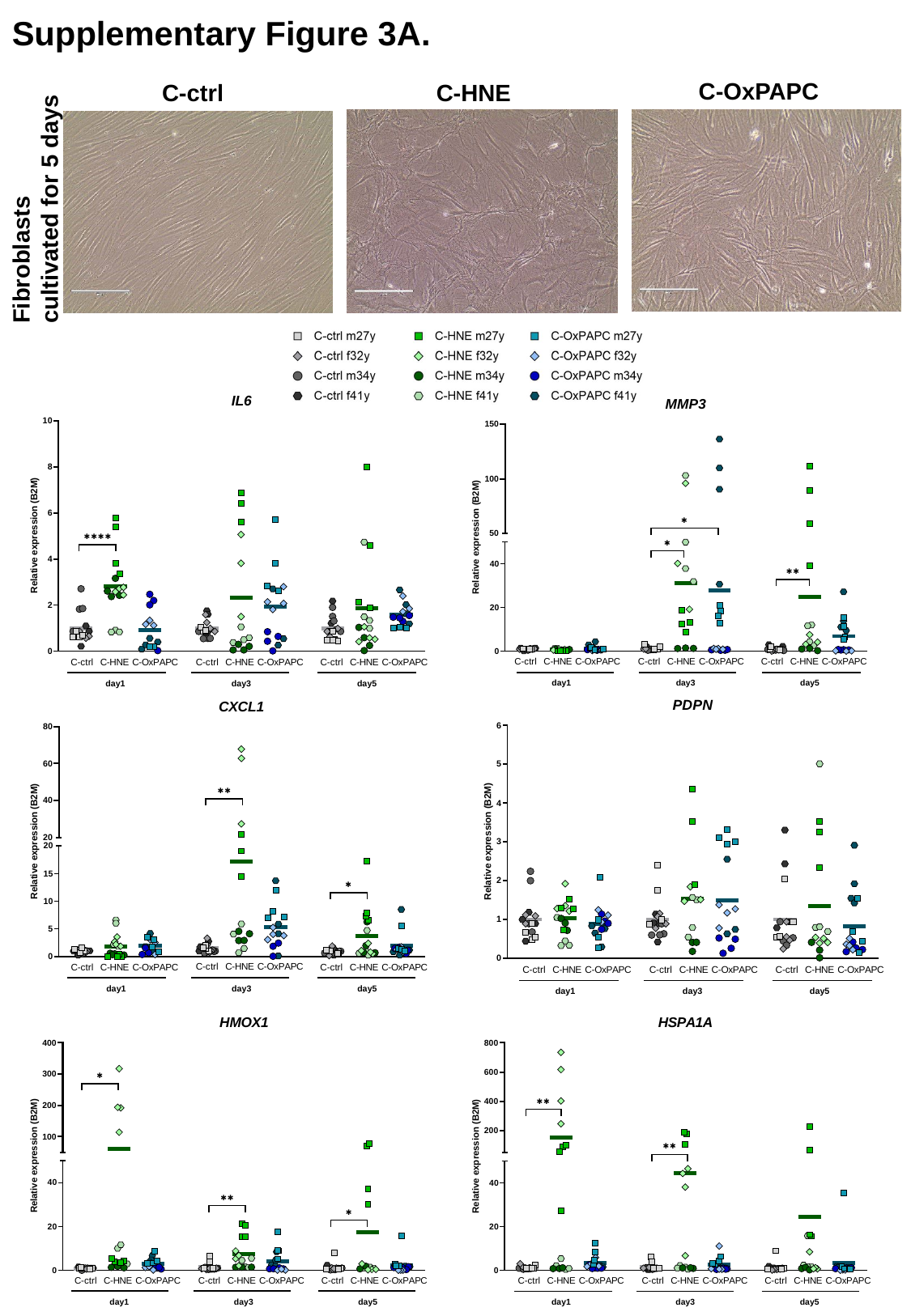

Supplementary Figure 3A.
C-OxPAPC
C-ctrl
C-HNE
Fibroblasts cultivated for 5 days

## Slide 4
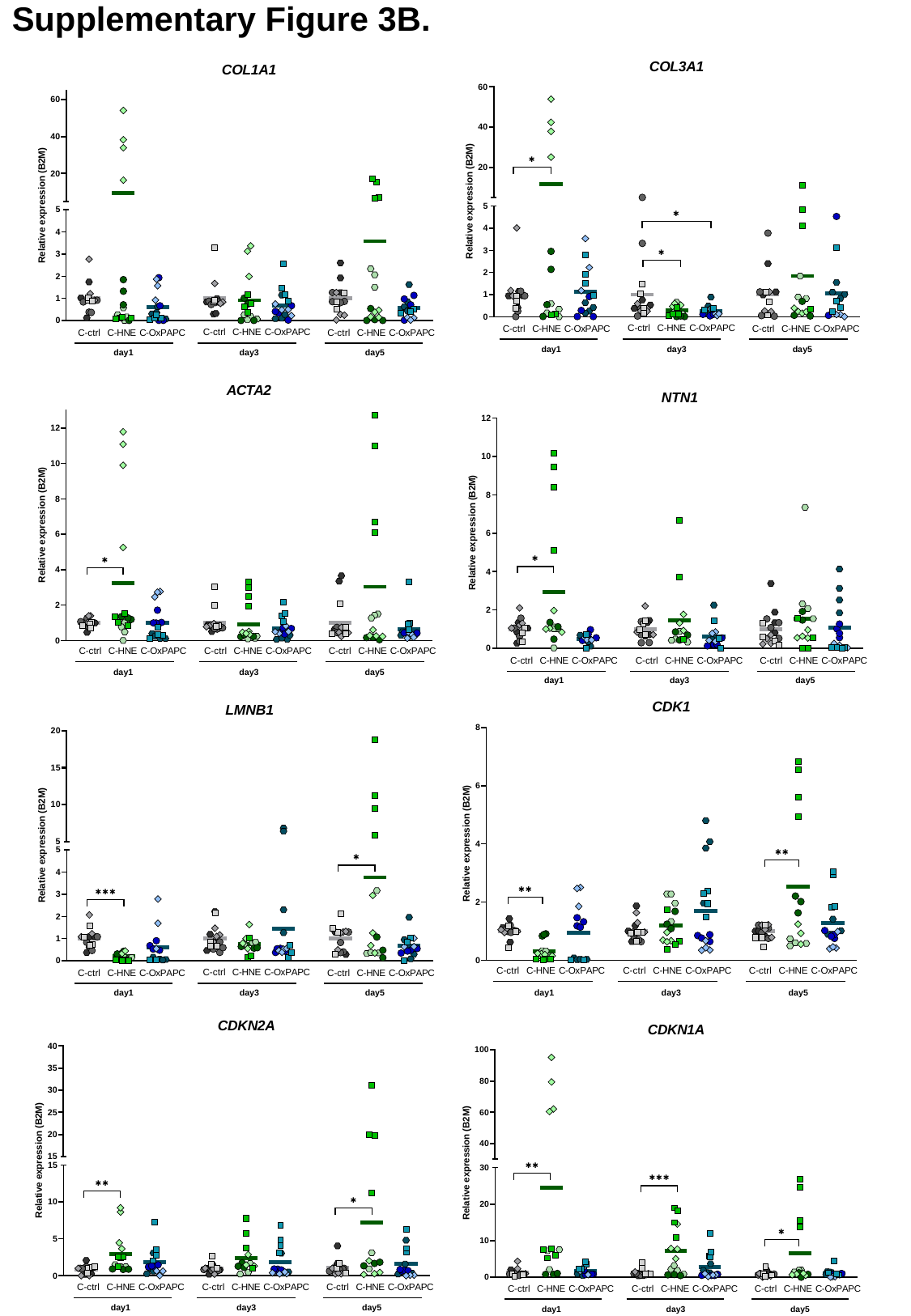

Supplementary Figure 3B.

## Slide 5
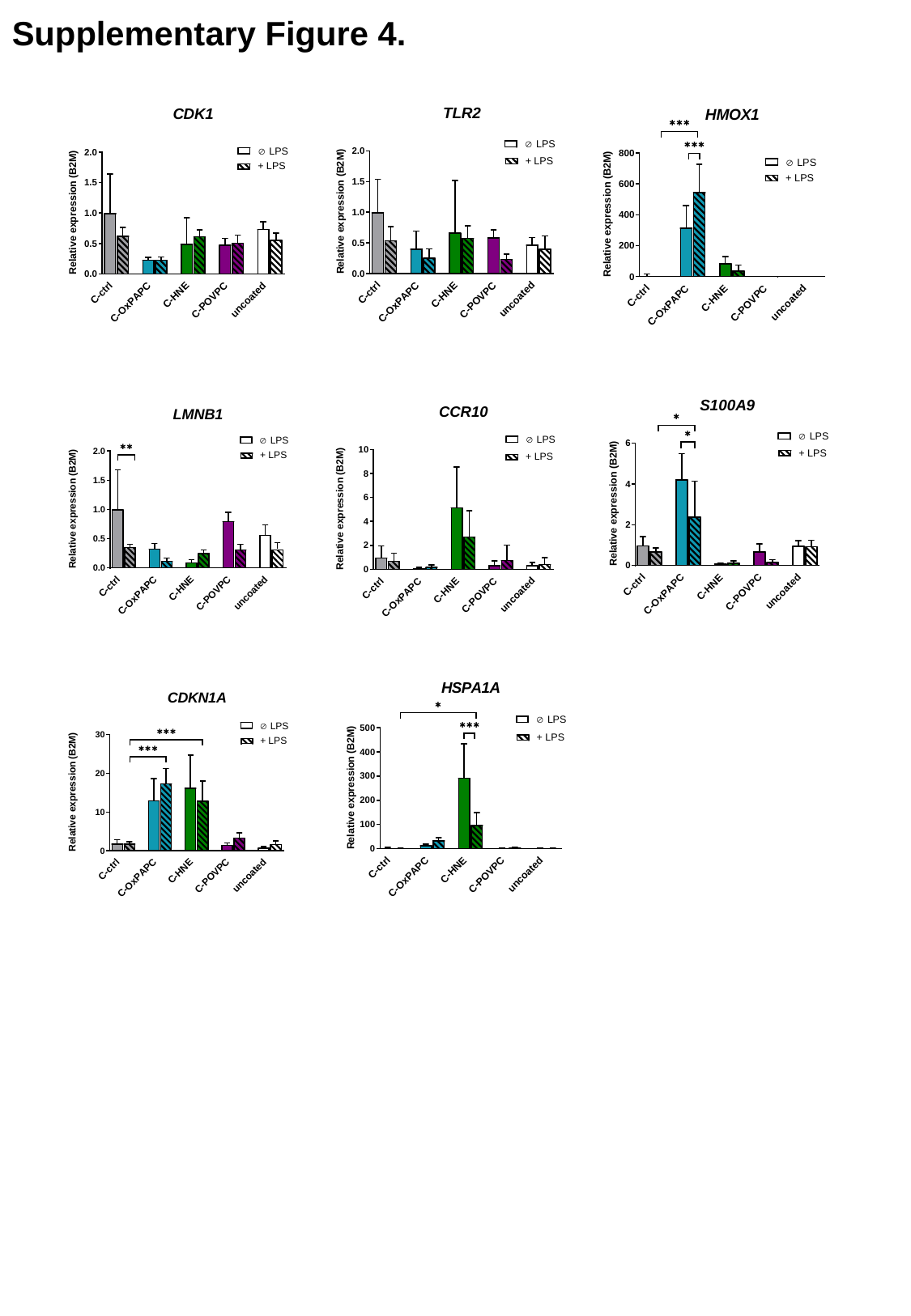

Supplementary Figure 4.

## Slide 6
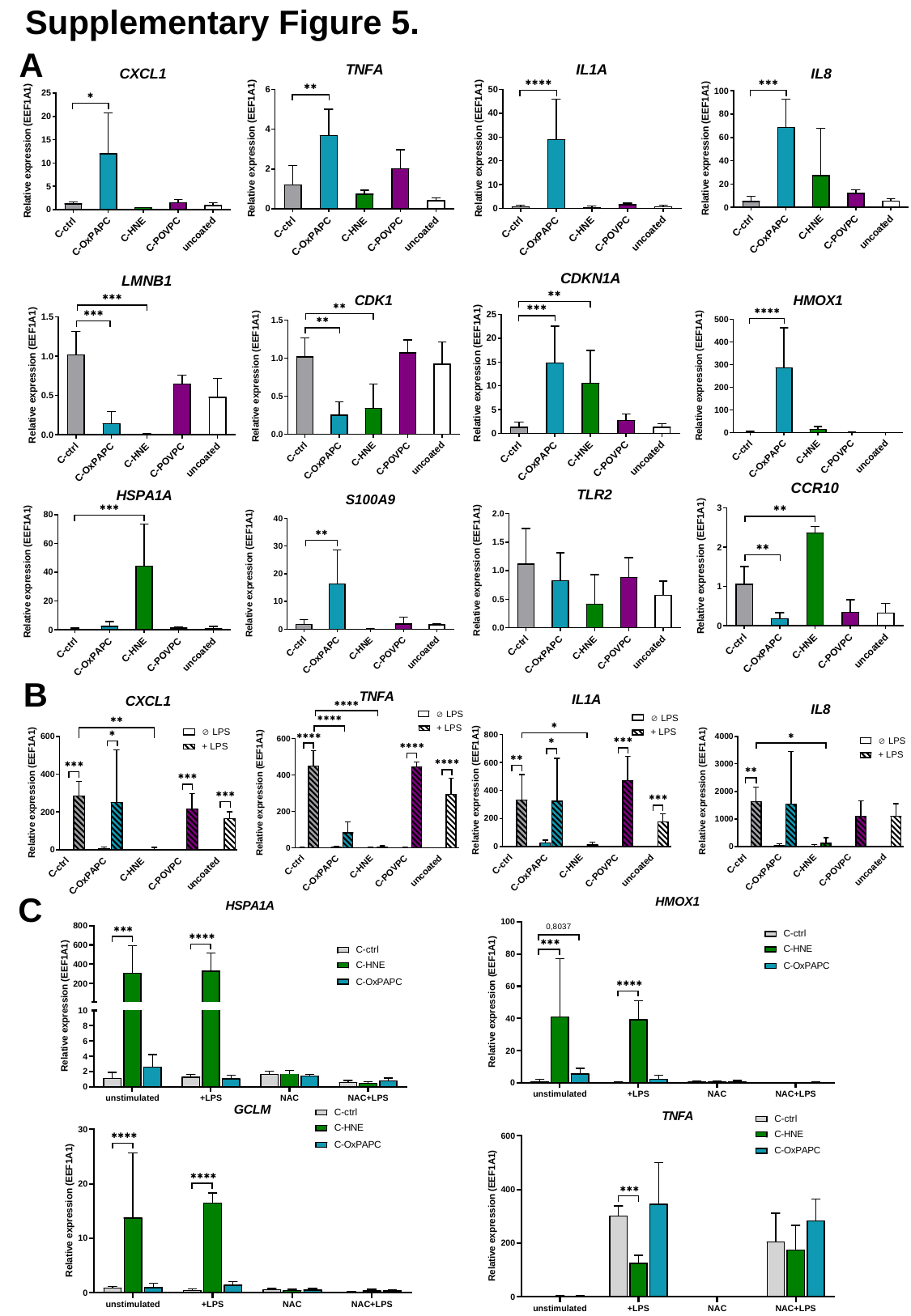

Supplementary Figure 5.
A
B
C

## Slide 7
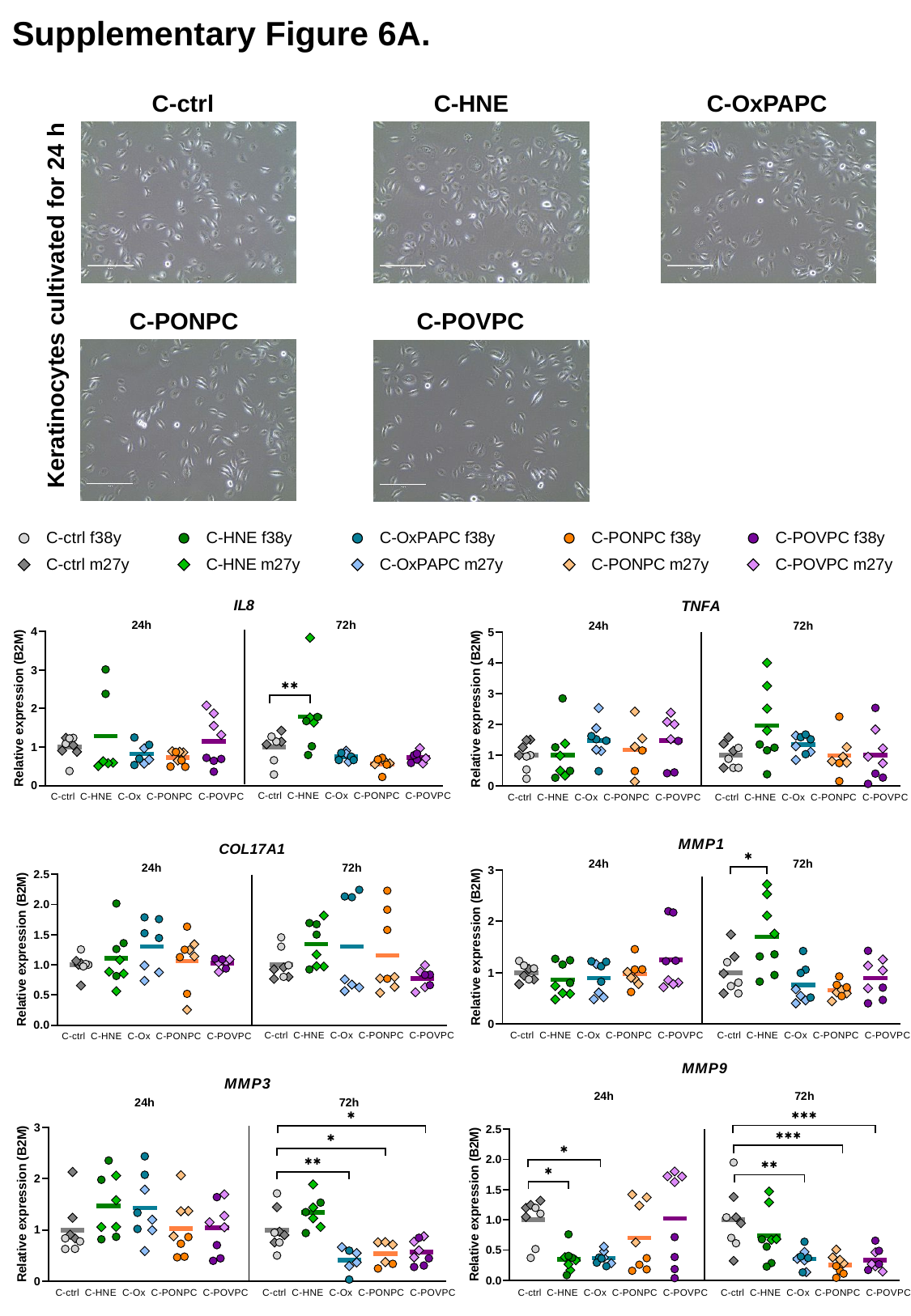

Supplementary Figure 6A.
C-OxPAPC
C-ctrl
C-HNE
Keratinocytes cultivated for 24 h
C-PONPC
C-POVPC

## Slide 8
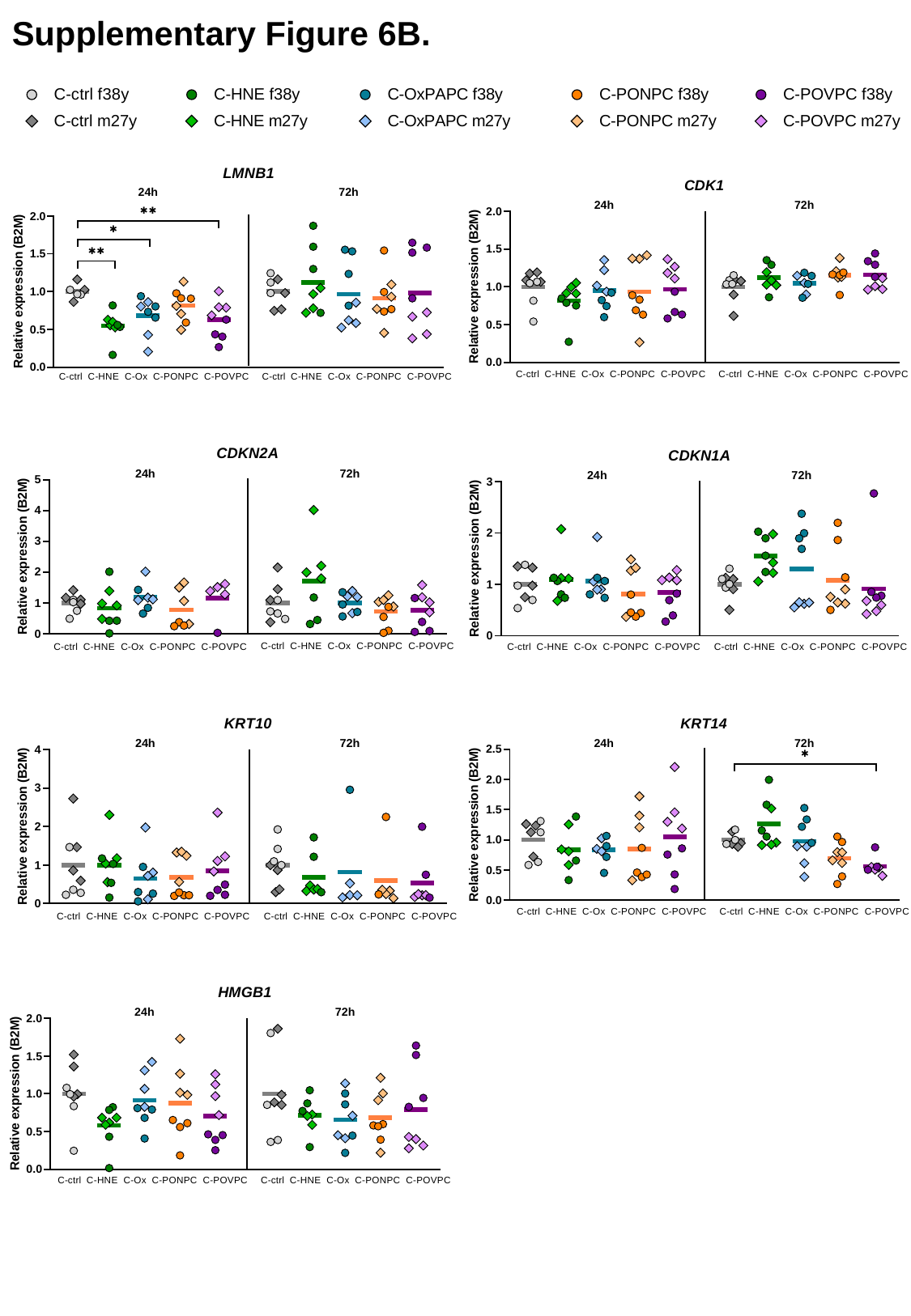

Supplementary Figure 6B.

## Slide 9
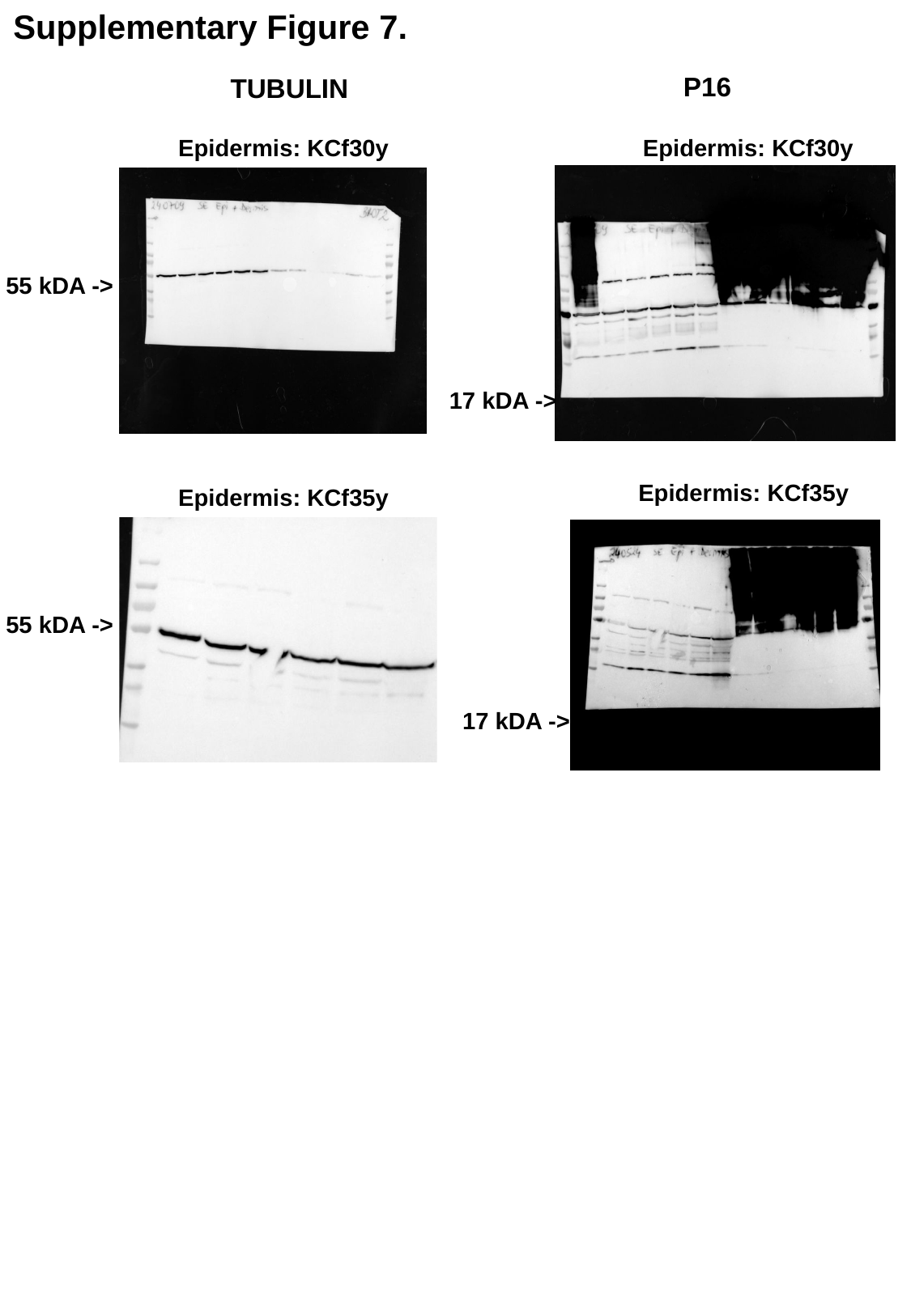

Supplementary Figure 7.
P16
TUBULIN
Epidermis: KCf30y
Epidermis: KCf30y
55 kDA ->
17 kDA ->
Epidermis: KCf35y
Epidermis: KCf35y
55 kDA ->
17 kDA ->

## Slide 10
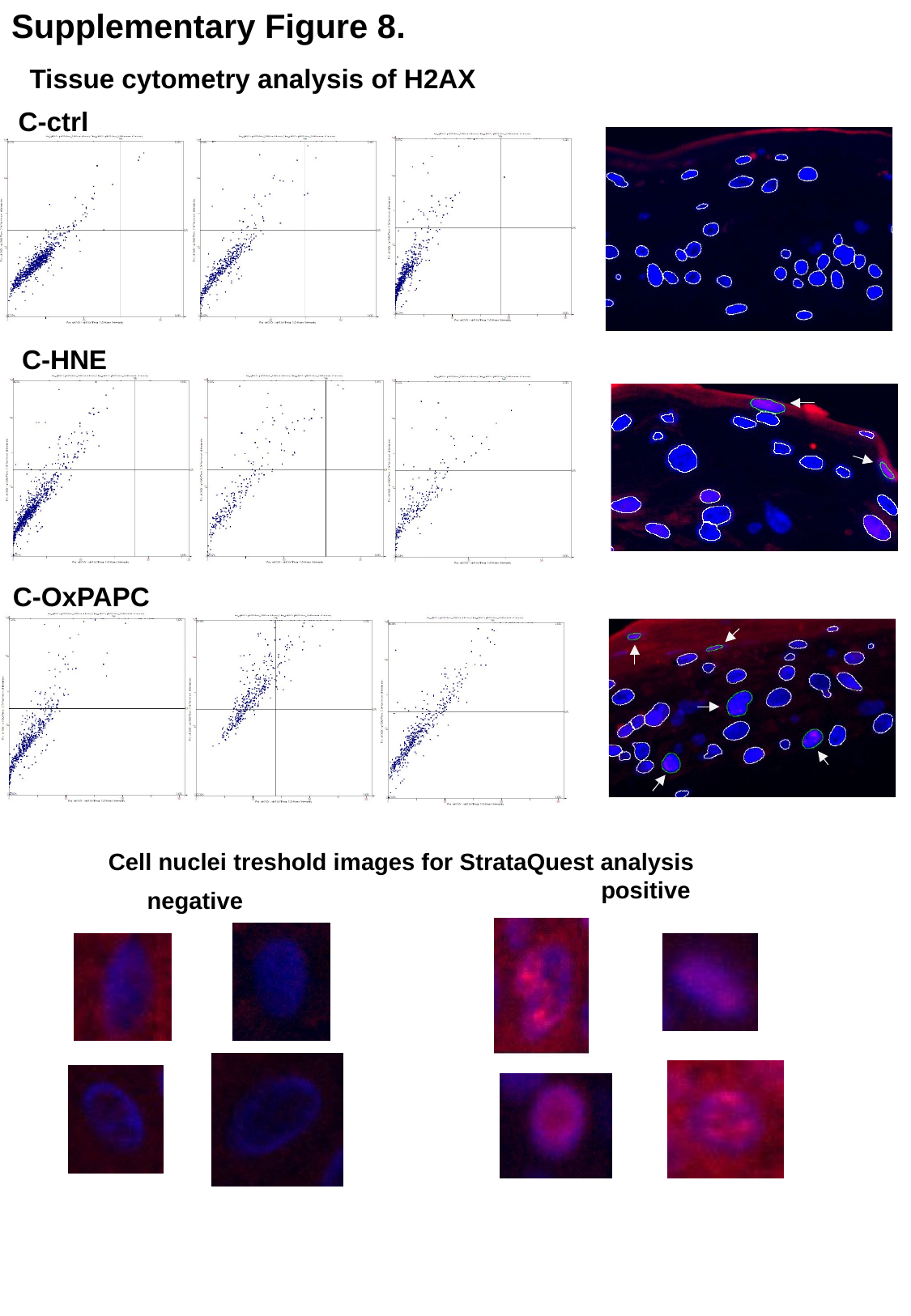

Supplementary Figure 8.
C-ctrl
C-HNE
C-OxPAPC
Cell nuclei treshold images for StrataQuest analysis
positive
negative
